# Classifying soft self-assembled materials via unsupervised machine learning of defects

**DOI:** 10.1038/s42004-022-00699-z

**Published:** 2022-07-14

**Authors:** Andrea Gardin, Claudio Perego, Giovanni Doni, Giovanni M. Pavan

**Affiliations:** 1 Department of Applied Science and Technology, Politecnico di Torino, Torino, Italy; 2 Department of Innovative Technologies, University of Applied Sciences and Arts of Southern Switzerland, Lugano-Viganello, Switzerland

**Keywords:** Coarse-grained models, Computational methods, Self-assembly, Supramolecular polymers, Characterization and analytical techniques

## Abstract

Unlike molecular crystals, soft self-assembled fibers, micelles, vesicles, etc., exhibit a certain order in the arrangement of their constitutive monomers but also high structural dynamicity and variability. Defects and disordered local domains that continuously form-and-repair in their structures impart to such materials unique adaptive and dynamical properties, which make them, *e.g*., capable to communicate with each other. However, objective criteria to compare such complex dynamical features and to classify soft supramolecular materials are non-trivial to attain. Here we show a data-driven workflow allowing us to achieve this goal. Building on unsupervised clustering of Smooth Overlap of Atomic Position (SOAP) data obtained from equilibrium molecular dynamics simulations, we can compare a variety of soft supramolecular assemblies *via* a robust SOAP metric. This provides us with a data-driven “defectometer” to classify different types of supramolecular materials based on the structural dynamics of the ordered/disordered local molecular environments that statistically emerge within them.

## Abstract (web summary)

Defects and disordered local domains in soft, self-assembled aggregates determine their dynamic and adaptive properties, and enable communication between entities, but characterizing and classifying such intricate dynamic behaviors is highly complex. Here, the authors report on a data-driven workflow to identify objective criteria for the comparison of complex dynamic features in soft supramolecular materials, deriving a data-driven ’defectometer’ that allows to classify soft self-assembled materials based on the structural dynamics of the ordered/disordered molecular environments that statistically emerge within them.

## Introduction

Supramolecular structures, composed of molecular units that self-assemble *via* non-covalent interactions, represent the key substrate for biological systems (membranes, micelles, protein fibers, etc.), and for new types of self-healing, stimuli-responsive and bioinspired materials^1–4^. Among many interesting features, the highest potential of soft supramolecular materials lies in their intrinsically dynamic character at room temperature^5^. The self-assembled monomers continuously exchange within and in-and-out these materials^6,7^, controlling how they communicate with each other at the equilibrium^8,9^, how they respond to external stimuli^10^, and how they behave out-of-equilibrium^11^. Such dynamical features determine a set of intriguing properties that, on the one hand are crucial for the functioning of biological tissues (e.g., self-healing, chemotacticity, molecular transport, etc.)^12–16^ and, on the other hand, are promising features for the design of new functional materials and nano-technologies^1,17–23^.

**Fig. 1:**
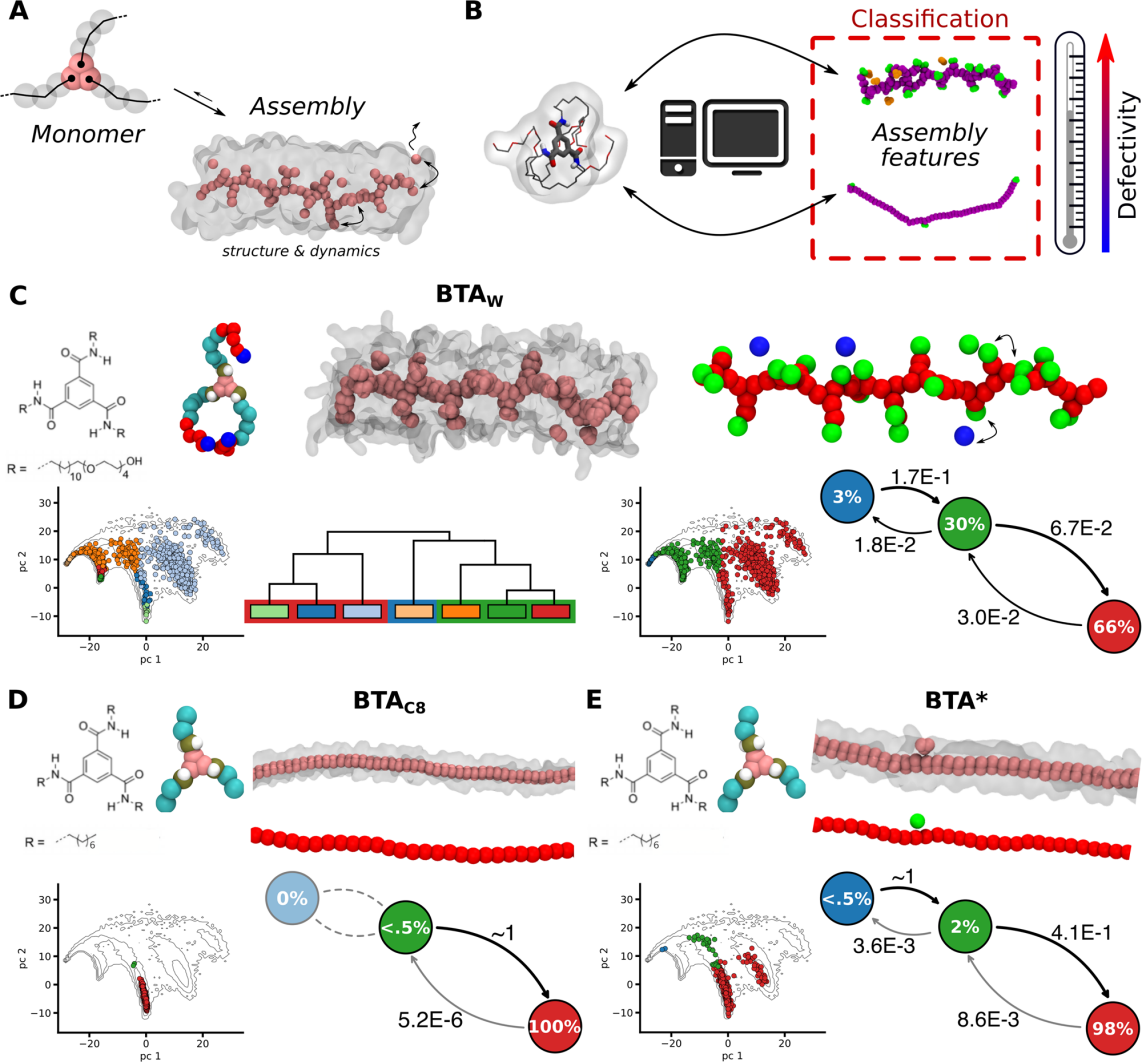
Classification of supramolecular polymers based on the structural/dynamical features of their molecular motifs. **A** Scheme showing an example of monomer self-assembling into supramolecular 1D fibers having a characteristic level of order/disorder which determines its structural/dynamical features (e.g., BTA)^6,7,34^. **B** A key question is how to design the monomers to obtain assemblies with controlled features. While ML techniques can be useful to this end, a first necessary step is developing a classification approach to compare different supramolecular structures in an unbiased way. **C** Analysis of the water-soluble BTA_*W*_ fiber. Top-left: chemical structure and CG model of BTA_*W*_ monomers, and equilibrium CG-MD snapshot of a BTA_*W*_ fiber^6,7,34^. Bottom-left: PCA scatter-plot of molecular motifs identified *via* SOAP + PAMM (projected on the first two PCs: PC1 and PC2). Each color corresponds to a different motif detected in the BTA_*W*_ fiber (the underlying contour plot shows the distribution of all SOAP vectors sampled in the three compared BTA fibers). The dendrogram indicates how the various detected motifs (microclusters: smaller rectangles) relate to each other, and how these can be merged into higher-level motifs (macroclusters: red, blue, and green larger rectangles). Top-right: coloring of monomer centers in an example equilibrium CG-MD snapshot based on the identified macroclusters. Bottom-right: PCA scatter-plot projection for BTA_*W*_ colored based on the identified macroclusters. Right: dynamic interconversion diagram, reporting the relative probability of transition between various macrostates, and the relative state population. **D**, **E** Analogous SOAP + PAMM characterization for the BTA_*C*8_ (**D**) and BTA^*^ (**E**) fibers based on the identified macroclusters.

A major goal in the study of self-assembled architectures is understanding how changes in the structure of the self-assembling building blocks (input) affect the overall properties of the supramolecular assembled architecture (output) (Fig. 1). Gaining such knowledge would pave the way toward the rational design of new functional materials with controlled properties, reducing the costs of trial-and-error synthesis. In the last two decades many efforts have been made in this direction. Notably, previous works thoroughly investigated and tried to rationalize how geometric properties of dispersed colloidal particles drive their mutual assembly into complex structures^24–27^. However, because of the complexity and the dynamical, multiform nature of such systems^28–31^, a direct connection between the collective properties of supramolecular materials and the microscopic features of the constituent monomers remains often impossible to attain.

Molecular simulations play a fundamental role in this framework, providing abundant data on the structure and dynamics of supramolecular assemblies, with thorough chemical-physical information on the factors that control the dynamical features of these materials^6,7,11,32–36^. However, analysing and interpreting the high-dimensional, high-detail data produced by computer simulations can be challenging, particularly for supramolecular systems. In such complex structures, irregularities and defects play a crucial role^6,8,10,11,16^, demanding for molecular descriptors capable to translate the simulation data into more reliable classifications of the molecular structure and dynamics characteristic of these systems.

"Human-based” analyses rely on low-dimensional descriptors, defined based on the experience and on those features directly readable by visual inspection. This often leads to biased predictions, affected by the choice of such low-dimensional descriptors, which possibly overlook important degrees of freedom of the system. To overcome these limitations, data-driven, Machine-Learning (ML) approaches, such as, e.g., unsupervised clustering, dimensionality reduction, pattern recognition, etc., are particularly useful to characterize complex self-assembling systems^37–41^. These techniques allow to fully exploit and analyse the rich, high-dimensional information contained in high-resolution MD simulations, providing insight on key patterns and correlations that may remain hidden to conventional human-based analyses^39,40,42–44^.

In particular, data-driven analyses allow to effectively monitor the local environment of each atom/molecule in a system, providing accurate, high-dimensional atomic/molecular descriptors, called “fingerprints”, that classify the mutual arrangement of atomic/molecular entities ^45–49^.

The Smooth Overlap of Atomic Position (SOAP)^49^, proved to be a very efficient density-based descriptor. It encodes the information of atomic environments into a rich, high-dimensional and agnostic (i.e., independent from a priori knowledge of the system) fingerprint^50–52^. Coupling a descriptor-based analysis with unsupervised clustering techniques enables a robust characterization of the ordered/disordered arrangement in molecular systems, as demonstrated for Lennard-Jones clusters, peptides^51^, and water^53–55^. Recently, combining SOAP descriptors and unsupervised clustering, we were able to reconstruct the complex structural dynamics of molecules in supramolecular materials, tracking e.g., defects formation and evolution^7^. This data-driven approach, in principle enables a rigorous and unbiased classification of soft structures^56,57^.

In the present work we push the data-driven comparability between supramolecular assemblies to the limit. Based on SOAP description and unsupervised clustering, as well as on a SOAP-based metric, we construct a data-driven analysis strategy, which can be used as a “defectometer”, measuring and comparing different structures based on their local molecular environments. This allows us to compare different types of assemblies, such as supramolecular fibers, micelles, layers, nanoparticles, etc. We obtain a general, unbiased classification of supramolecular systems that are profoundly different among each other, capturing structural and dynamical aspects that are hardly readable with standard descriptors.

## Results and discussion

The formation and dynamics of defects play a fundamental role in determining the properties of soft, supramolecular assemblies. Detecting and classifying such structural defects require adequate descriptors of local molecular environments, carrying high-dimensional information on the monomer arrangements and dynamicity. In this framework, general, data-driven descriptors and analyses were recently proven to be more effective than standard, “human-based” descriptors, especially for complex and dynamic molecular systems^7^. In the following, we characterise the behavior of different types of soft, supramolecular self-assembled materials as emerging from equilibrium MD trajectories obtained using CG molecular models. For all considered systems, we compute the SOAP spectra associated to the local molecular environment that surrounds each monomer core in the system, at every sampled step of an equilibrium CG-MD trajectory. Gathering these SOAP spectra in global datasets, we can compare and classify different systems. (i) Combining dimensional reduction and unsupervised clustering techniques, we can determine from the SOAP dataset the main molecular motifs present in each studied assembly. This allows us, for example, to identify defects in such soft assemblies that are key for their intrinsic dynamics^6–8^. (ii) By defining a SOAP-based metric, we can compute the distance between the compared systems in the high-dimensional SOAP space, assessing and quantifying their similarity in terms of the SOAP monomeric motifs that emerge and are present within them, in equilibrium conditions^55,56^. This approach is first applied to a set of one-dimensional supramolecular polymers, having different monomer structures, and is then extended to compare and classify two-dimensional as well as three-dimensional assemblies with each other, proving a remarkable flexibility.

### Comparing variants of a supramolecular polymer

Recently, it has been shown that unsupervised clustering of SOAP data from equilibrium MD of 1,3,5-Benzenetricarboxamides (BTA) supramolecular polymers allows the reconstruction of the structural dynamics of such complex assemblies^7^. In particular, monitoring the SOAP vectors defined in the center of each monomer in a BTA supramolecular fiber allows us to retrieve detailed information on the structural order/disorder (both at a local and global level), as well as on the internal monomer dynamics that characterize such supramolecular polymers. A pre-requisite is that the MD simulation trajectories considered in the analysis provide sufficient sampling of the equilibrium of the simulated systems (in terms of, e.g., microstates, their populations, and transitions between them). All simulations used in the following analyses meet such requirement (see the Methods section for complete details on the simulation and analysis protocols).

Considering different variants of BTA fibers, and combining the SOAP data obtained for each system, we can assemble a dataset gathering all possible configurations (each one identified by a SOAP feature vector) visited by monomers in the different BTA fiber variants. This guarantees that SOAP vectors in this dataset, associated to the local arrangement of monomers in all the different assembly variants, belong to a unique high-dimensional feature space, thus allowing us to compare between different systems^7^. In these analyses, the resolution of the employed molecular models is crucial, as it implicitly determines the accuracy by which, e.g., the monomer-monomer interactions, the monomers’ flexibility, etc., are described. Coarse-grained (CG) models with a resolution <5 Å— developed based on the Martini force-field^58^, and optimized to match the behavior of the respective all-atom (AA) models—were proven accurate enough to capture all essential information while guaranteeing sufficient sampling of the assembled fibers in equilibrium^6,7,34^. With such CG models, it was demonstrated that water-soluble BTA polymers, composed of BTA_*W*_ monomers with amphiphilic arms, possess a variegated structure with a rich and diverse set of monomeric states, in continuous exchange and dynamic interconnection with each other. The SOAP data of the BTA_*W*_ fiber were analysed *via* the Probabilistic Analysis of Molecular Motifs (PAMM) unsupervised clustering (see Methods section for details)^7,51^. As shown in Fig. 1C, PAMM identifies different molecular motifs (microstates) in the SOAP data, indicated by different colors in the 2D PCA projection. The hierarchical structural/dynamical interconnections between microstates (i.e., how structurally similar they are, and how much they dynamically exchange with one another) are captured by a statistical analysis of the identified microclusters (see the Methods section) which outputs the related microstates dendrogram (Fig. 1C, bottom-left). As the SOAP data analyzed herein are extracted from snapshots taken along equilibrium MD trajectories, the microclusters that are found close to each other in the dendrogram identify SOAP monomer environments (monomer microstates) that are similar from a structural point of view (see, e.g., Supplementary Fig. 5) and/or quickly dynamically exchanging with each other. The dendrogram also allows to perform a systematic coarse-graining of the classification, identifying the dominant motifs (macrostates) in the BTA_*W*_ fiber (Fig. 1C, right): the ordered/persistent interior (bulk) of the fiber (in red: ~66% of the monomers), the stacking defects (~30% of the monomers, in green: bound to the stack only by one side), and the monomers which are adsorbed on the fiber surface, moving from one defect to another (in blue: population ~3% of the monomers). From the frequency at which the monomers change state during the equilibrium MD, we can estimate the relative transition probabilities between the various states. The BTA_*W*_ fiber turns out to possess a very rich and diverse internal dynamics. In comparison, the BTA_*C*8_ variant, with shorter carbon side-chains, produce straight and substantially defect-free fibers (Fig. 1D). Interestingly, artificially reducing the directional interactions between the monomers a new BTA^*^ fiber variant is defined, where a fraction (~2%) of monomers form defects that are continuously created-and-repaired ^7^.

It is worth noting that such a direct comparison between fiber variants is possible since all SOAP vectors, sampled in the compared systems, belong to the same high-dimensional feature space (324-dimensional, see Methods for details). This approach allows to identify defects (i.e., disordered domains) in the supramolecular fibers, to track their emergence/disappearance, and to use them to compare fiber variants. Moreover, as the concept of defects is elusive in such dynamical structures, this approach is general and flexible, as the definition of the molecular motifs that statistically populate the assemblies, and thus of defects, is exquisitely data-driven. This analysis can be thus extended to other families of assemblies. As a first proof-of-concept, we proceed by comparing variants of different supramolecular polymer families.

**Fig. 2:**
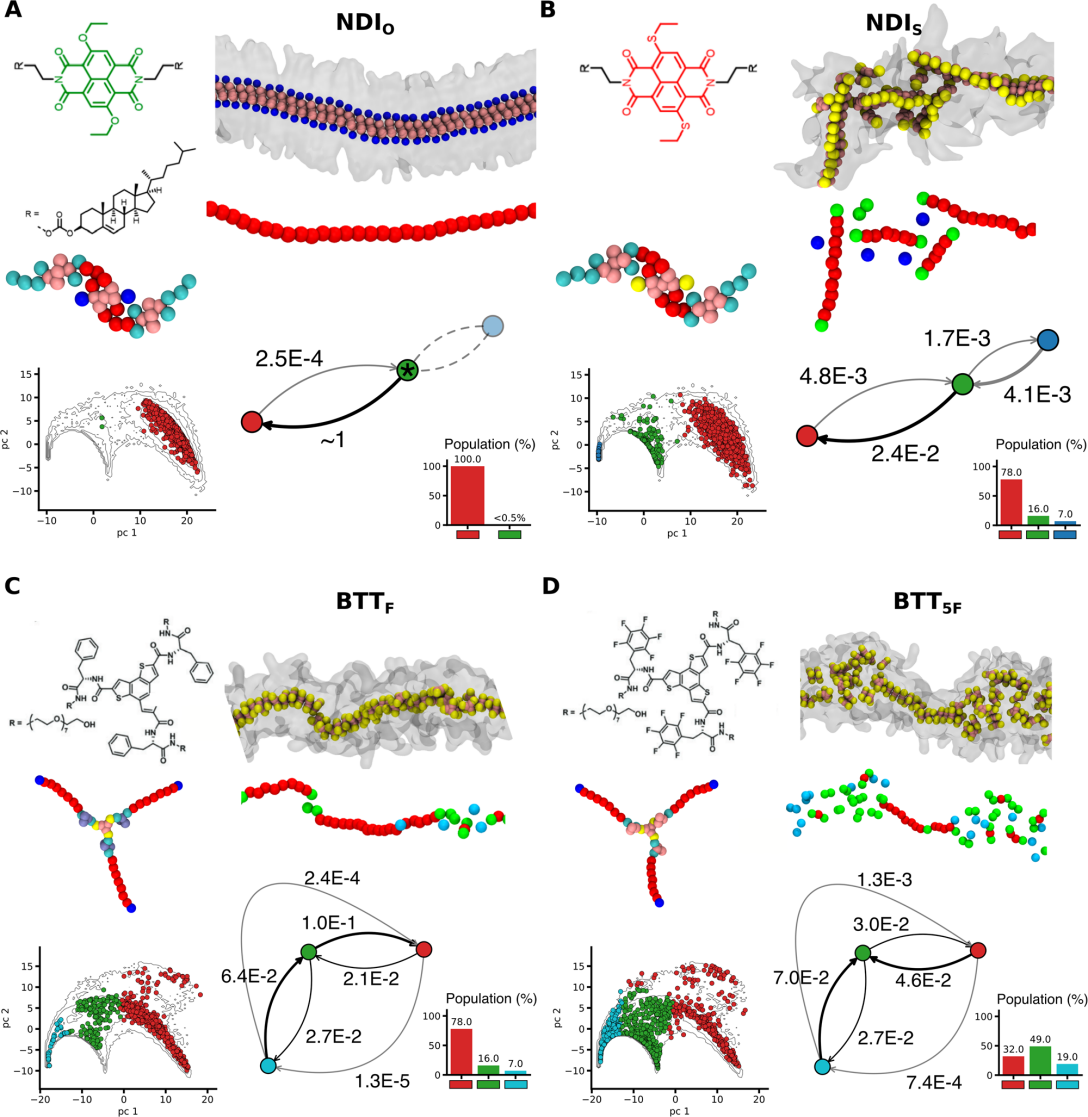
Comparing variants of supramolecular polymers. **A**–**D** SOAP and PAMM analysis on NDI and BTT supramolecular polymer variants: (**A**) NDI_*O*_ monomer, (**B**) NDI_*S*_ monomer, (**C**) BTT_*F*_ monomer, and (**D**) BTT_5*F*_ monomer. Each panel summarizes the results of the analysis for a single system variant, showing (from top-left): chemical structure and CG model of the monomer; equilibrium CG-MD snapshot of the assembly, and view of the same with monomer centers colored according to the identified molecular motifs; scatter-plot showing the PCA (projected on the first two PCs) of SOAP descriptors, colored according to the motif. Interconversion diagram and population histogram. Asterisks indicate rare fluctuation states, with residence time ~0 (e.g., green cluster in NDI_*O*_).

We focus on two other families of supramolecular polymers, formed by monomers based on naphthalene diimide (NDI)^59^ (Fig. 2A, B) and benzotrithiophen cores (BTT)^60^ (Fig. 2C, D). We compare two variants per-family, NDI_*O*_ vs. NDI_*S*_, and BTT_*F*_ vs. BTT_5*F*_, for which we employed reliable CG models having analogous resolution of the BTA models used in the analyses of Fig. 1^59,60^. We repeated the analysis performed for BTAs to compare the NDI_*O*_ and NDI_*S*_ fiber variants, building a unique SOAP dataset from the equilibrium MD of the two systems (Fig. 2A, B). Also in this case, we computed SOAP vectors, defined in the center of each monomer (see Supplementary Fig. 11 for a visual representation), over equilibrium MD trajectories, using the same sampling frequency and length used for BTA analysis (see Methods section for complete details). We then performed PCA on the SOAP dataset, and used PAMM to identify the molecular states. Again, in this case, PAMM highlighted three dominant monomeric states (Fig. 2A, B: in red, green and blue). Indeed, this analysis of NDI variants evidences a picture similar to that of BTAs. One variant, NDI_0_, exhibits a well-ordered structure with all monomers belonging to the fiber backbone (Fig. 2A: in red), apart from rare fluctuations. The slight change in the NDI_*S*_ monomer structure (the replacement of two oxygen with two sulfur atoms) produces instead a more disordered fiber, rich of defects (Fig. 2B, in green: ~16%), and of adsorbed monomers which travel from one defect to another within the assembly (in blue: ~7%). The same protocol was used for the BTT fibers (Fig. 2C, D), showing again three molecular states, and providing a similar picture to that of BTA and NDI systems. The BTT_*F*_ molecule tends to induce a much ordered fiber than the BTT_5*F*_ variant, in agreement with what is known experimentally for these systems^60^. A difference that emerges from these analyses, is that BTT fibers possess a more diverse structure than NDI fibers, which appear as more ordered in comparison. In particular, BTT_5*F*_ exhibits the highest occurrence of disordered monomeric domains (~49% of the monomers are in defected state and ~19% are adsorbed/traveling monomers). The ordered monomers in the BTT_5*F*_ fibers are only ~32% (~78% in BTT_*F*_), and considerable dynamic interconversions are observed between all monomeric states (Fig. 2C, D).

The proposed analysis allowed us to qualitatively detect the statistical formation of relevant molecular motifs such as defects. This is an important ingredient since, as demonstrated for BTA supramolecular polymers^6–8^, the possibility to create and repair defects in the assembly is crucial for their dynamic properties^10^. The analyses of Figs. 1, 2 demonstrate that this approach is versatile, and it can be used to compare fiber variants within the same supramolecular polymer family. However, at this stage, the analysis does not allow us to unambiguously compare between the different families. Especially, since the definition of defects emerge from the data contained in separate SOAP datasets, it is not clear to what extent the defects in the BTA fibers are comparable to the BTT or NDI ones. The SOAP dataset used to compare the BTA variants in Fig. 1, in fact, does not contain information on the monomer states in the NDI and BTT fibers. To compare these supramolecular fibers in a more objective and quantitative way, a further step is required.

### Comparing different types of 1D supramolecular polymers

As described above, by processing a comprehensive dataset containing SOAP vectors sampled via MD simulation of multiple systems, one can build a framework to rigorously and unambiguously compare different supramolecular systems. The idea is to retain in the SOAP analysis those relevant features that are common to all the systems (a sort of “common molecular denominator”)^7^. As detailed in the Methods section, we considered only the position of monomer centers in the SOAP calculation, with two advantages: (i) this has been shown to retain sufficient information on the monomer arrangement in the fibers, and thus on their supramolecular structure and dynamics^7,8^. (ii) This makes the analysis very general/abstract—all assemblies are composed of mutually interacting monomers—opening the possibility to compare, not only variants of a supramolecular fiber, but also widely different assemblies.

**Fig. 3:**
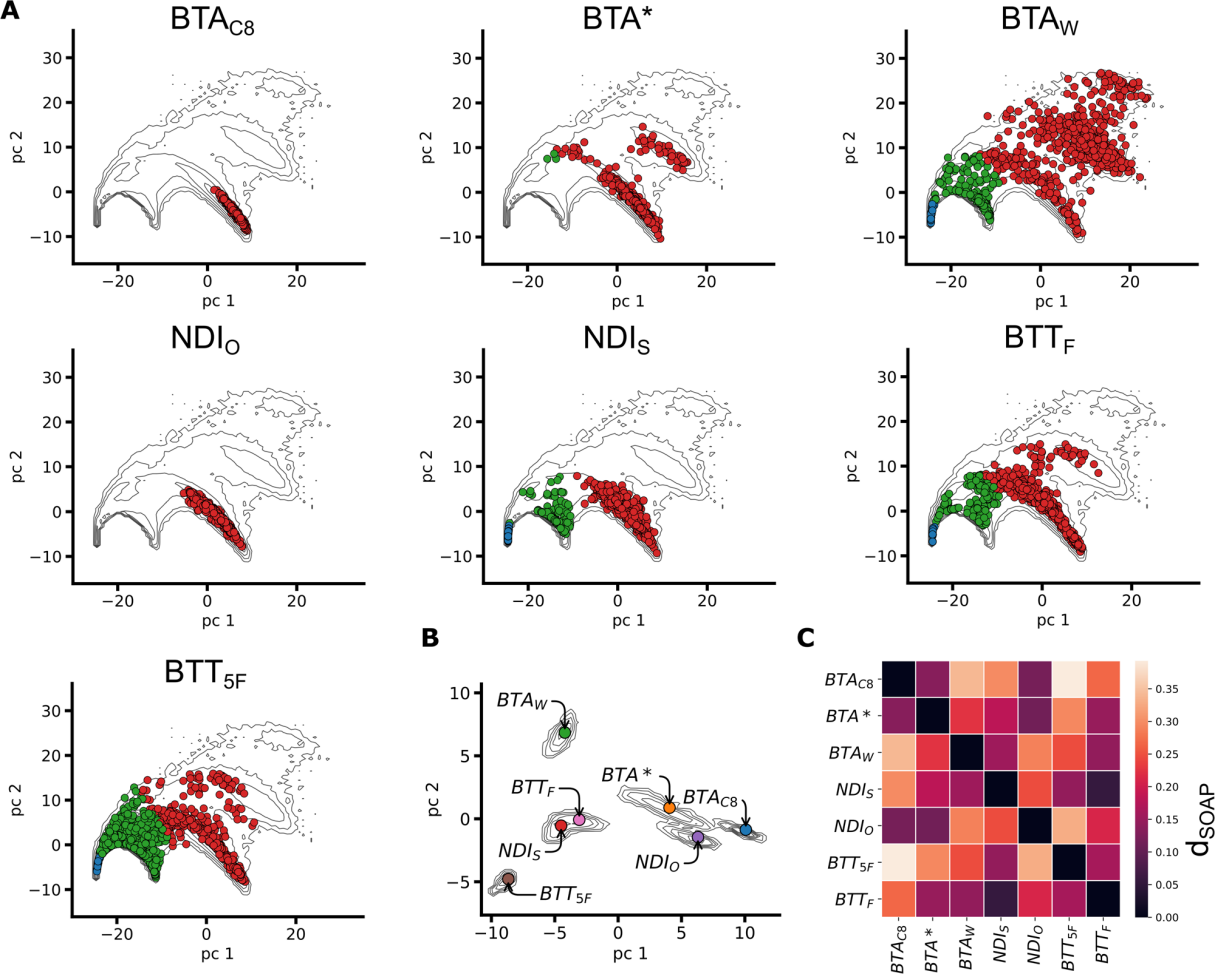
Comparing 1D assemblies. **A** Scatter plots of molecular motifs (macroclusters) detected for the different supramolecular polymers analysed. The PCA projected on the first two PCs (PC1 and PC2) of the of SOAP vectors are shown, colored based on the identified (PAMM) macroclusters. The scatter plots are embedded in a global contour plot of the complete dataset (SOAP vectors of all the seven 1D supramolecular fibers). **B** Contour plot of the *frame*-average distributions computed from the PCs of the SOAP vectors. The colored dots represent the PC projection of *simulation*-averages for each 1D fiber. This manifests the adjacency of different systems in terms of SOAP (although distortions might emerge from the projection on the PC1, PC2 plane). **C** Distance (*d*_SOAP_) matrix (left) built from the *simulation*-averages of the SOAP vectors (Eq. 7 in the Methods section). The color scale indicates the distance (*d*_SOAP_) between the assemblies (in terms of monomeric motifs present within them) defined in the 324-dimensional SOAP space, retaining the complete information of the SOAP descriptor.

To this end, we thus built a joint dataset containing all the SOAP vectors sampled *via* the equilibrium MD of the systems simulated for the analyses of previous section (3 BTA, 2 NDI and 2 BTT fibers), obtaining the global contour plot reported in Fig. 3A. We repeated the PCA and PAMM analyses over this dataset, identifying the main molecular motifs shared by all these one-dimensional assemblies (Fig. 3A). As in the analyses of Figs. 1, 2, three main structural clusters are identified by PAMM: backbone (in red), defects (in green) and adsorbed/surface-diffusing monomers (in blue). This distinction in three macrostates is used in the the PCA scatter plots of Fig. 3A to classify the SOAP states sampled by the individual systems, overimposed on the global SOAP distribution (contour plot). These scatter plots can represent characteristic “fingerprints” of the supramolecular structures, indicating which molecular states are populated in each system. Qualitatively, such fingerprints provide an information of similarity between systems, in terms of structural arrangement and dynamicity of the monomers. However, since PCA scatter plots are the result of a dimensionality reduction, they can provide a distorted picture, where dimensions potentially relevant in the overall feature space may be neglected.

To reach a more quantitative insight, we employed a SOAP-based metric that allows comparing complex supramolecular systems based on the average SOAP spectra of the monomers forming the assemblies (i.e., based on the structural/dynamical features of the local environment that, on average, surrounds the monomers). Proven useful to compare complex interacting molecular systems, such as, e.g., lipid bilayers^56^, or liquid aqueous systems^55^, we build on such high-dimensional metric to quantify the similarity between the SOAP data associated to each fiber (see Methods for details). For each frame extracted from an equilibrated MD simulation, SOAP vectors associated to the local environments surrounding all monomers are averaged into a single SOAP spectrum (the *frame*-average, Eq. 4). These frame spectra are then averaged along the MD trajectory, obtaining an average SOAP spectrum, which is characteristic of a given assembly and of those molecular motifs that populate it at equilibrium (the *simulation*-average, Eq. 5). The *frame*- and *simulation*-averages associated to each system are projected onto the first two PCs, in the scatter plot of Fig. 3B. We can thus assess the similarity among the different 1D assemblies in a more rigorous way, by employing a SOAP-induced metric^50,55,56^ (Eq. 7) to compute the distance (*d*_SOAP_) between the SOAP *simulation*-averages of the various systems. Such distance is defined directly in the SOAP space, and preserves the complete information contained in such high-dimensional descriptors. The result of this analysis is the *d*_SOAP_ distance matrix in Fig. 3C. The off-diagonal values in the *d*_SOAP_ matrix grant a classification of the assemblies under investigation in the global SOAP space. The darker the color of the entry, the lower is the *d*_SOAP_ between the assemblies, indicating their similarity (in terms of molecular environments). The *d*_SOAP_ matrix confirms the qualitative indications provided by the scatter plots of Fig. 3A: the most ordered supramolecular polymers, namely, BTA_*C*8_, BTA^*^, and NDI_*O*_, are similar and mainly populated by ordered, backbone-like molecular domains (Fig. 3A: in red). In these systems, the defect states are just sparsely populated, and located very close to the ordered domains—this is consistent with the fact that defects are relatively rare fluctuations, which are readily repaired (consistently with, e.g., Fig. 1E). The remaining 1D systems, containing a higher defect concentration (Figs. 1, 2), are more or less distant from the previous three fibers. NDI_*S*_ and BTT_*F*_ SOAP spectra are nearly superimposed, with a *d*_SOAP_ ~ 0, and very similar PCA projections (Fig. 3A, B). This indicates that the monomeric environments associated to these systems are, on average, structurally/dynamically very similar (see also population histograms and interconversion graphs in Fig. 2). Conversely, BTT_5*F*_ and BTA_*W*_ present unique features, distancing themselves from the other fibers. Such a peculiarity, already suggested by the fingerprints of Figs. 1, 2, is here confirmed quantitatively.

Classifying different types of supramolecular polymers, these results demonstrate how the proposed approach is effective in comparing 1D assemblies in general. This is possible through the unbiased, high-dimensional, SOAP-based metric, that quantitatively compares the average molecular environment (and defects) emerging in the various assemblies. The flexibility of the approach suggests to investigate whether such a “defectometer” can be used to compare assemblies with higher structural dimensionality (i.e., 2D or 3D assemblies).

### Comparing 2D dynamic assemblies

**Fig. 4:**
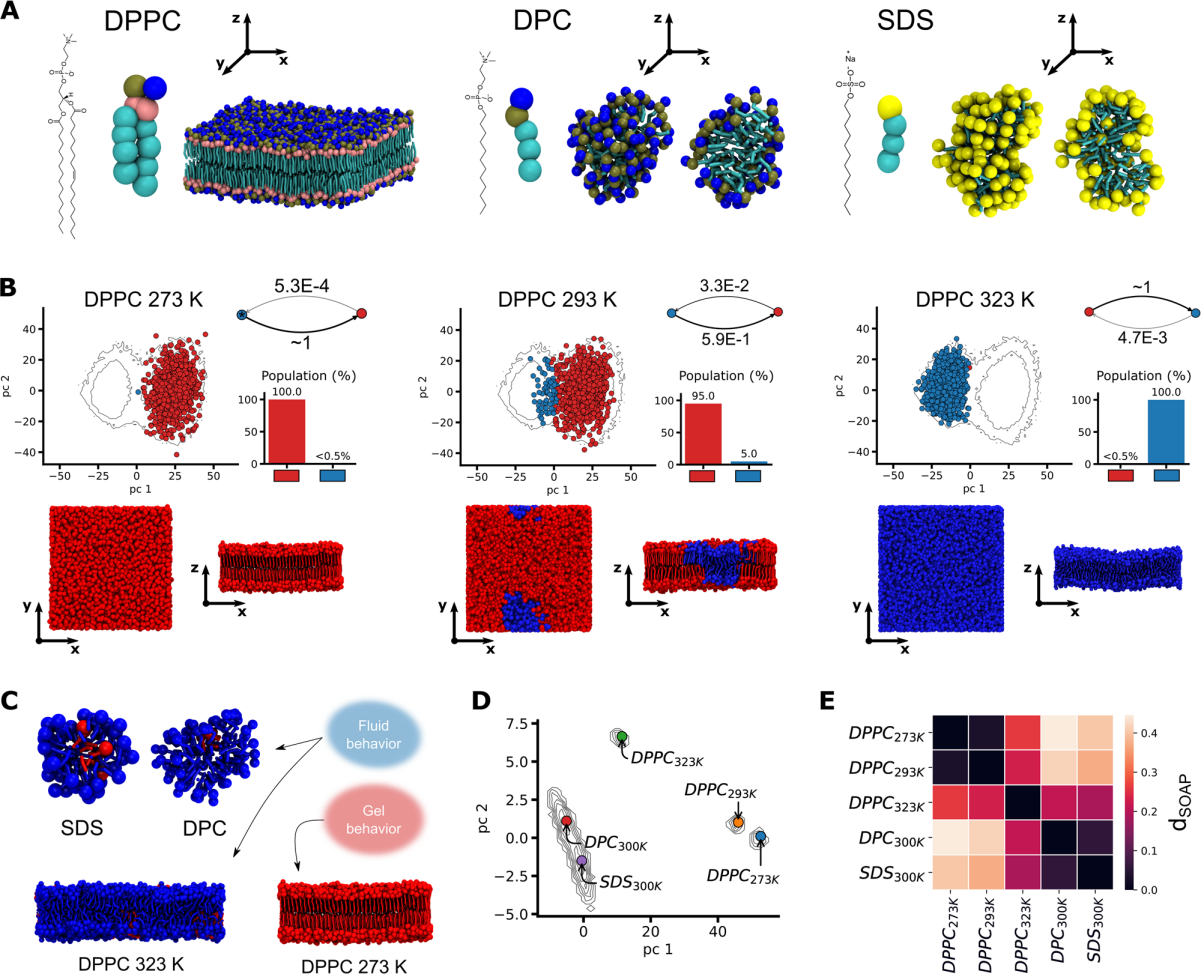
Comparing 2D assemblies. We compare assemblies where the monomers are displayed on flat (i.e., DPPC lipid bilayers) or curved/spherical surfaces ((i.e., DPC and SDS micelles). **A** Three example 2D assemblies (CG models for the monomers and their respective aggregates) considered herein. **B** SOAP + PAMM analysis for a DPPC lipid bilayer model at three different temperatures. For each value of *T* we report: the PC scatter plot of SOAP feature vectors (top-left), the molecular motifs (macroclusters) interconversion graph and population histogram (top-right), an equilibrium MD snapshot of the bilayer from top and side views (bottom). The scatter-plot circles and the lipids in the snapshots are colored according to the different motifs detected by PAMM analysis. **C** Equilibrium MD snapshots for each studied systems, with monomers colored according to the molecular motifs (macroclusters) detected in the analysis of the global dataset of the 2D aggregates (see Supplementary Fig. 10 for the individual PCA scatter plots). **D** Contour plot of the *frame*-average distributions, computed from the PCs of the SOAP vectors. The colored dots represent the *simulation*-averages for all considered assemblies in the PC reduced space. **E** Distance *d*_SOAP_ matrix for all 2D assemblies.

We here extend our approach to compare 2D supramolecular systems. We focus on assemblies where the monomers may be arranged on flat, as well as on curved surfaces. As case studies, we chose DPPC lipids, which self-assemble into 2D (planar) lipid bilayers, and DPC and SDS surfactants, that form nearly spherical micellar aggregates. For all these systems, we employed validated Martini-based CG models (same resolution of the previously studied models) and collected equilibrium MD trajectories for the SOAP analysis (Fig. 4A). Also in these analyses, we consider one SOAP center per-monomer (centered in the lipid/surfactant heads).

DPPC bilayers are known to undergo a gel-to-liquid transition around ~300–320 K of temperature, which is well-captured by DPPC Martini models^56^. In our analysis, we considered equilibrium MD trajectories for DPPC at *T* = 273, *T* = 293, and *T* = 323 K, as representative of 2D planar assemblies having variable reconfiguration dynamics of monomers, depending on the bilayer gel/liquid state. Analysing the DPPC trajectories at different temperatures, we first performed a SOAP + PAMM analysis, analogous to the SOAP + PAMM comparisons between fiber variants belonging to the same family, in Figs. 1, 2 (see Methods for details). The results prove that this approach captures the bilayer gel-to-liquid transition correctly, solely based on how the environment surrounding the lipids changes with the temperature (Fig. 4B). At low temperature (~273 K, Fig. 4B: left) the bilayer is entirely in the gel phase (in red), while as the transition temperature is approached (between ~300 and 320 K), liquid domains appear (in blue, ~5% at ~293 K, Fig. 4B: center)^56^. When the temperature raises to ~323 K, the bilayer is entirely liquid, with residual (<5%), gel-like lipids (Fig. 4B: right). Our analysis built on the SOAP data correctly distinguishes gel and liquid domains in planar lipid bilayers, without prior knowledge on the lipid arrangement in each phase. Nonetheless, interesting questions may arise on how similar the identified monomeric environments are to, e.g., those present on the curved surface of a micelle.

We thus, extended our SOAP + PAMM analysis to curved 2D assemblies, enriching the dataset with the SOAP data obtained from the equilibrium MD (at *T* = 300 K) of two micellar aggregates, made of DPC or SDS surfactants. The PCA scatter plots (Supplementary Fig. 10) indicate a significant overlap of both micelles fingerprints with that of the lipid membrane in the liquid phase (*T* = 323 K). This suggests that the structural/dynamical features of the monomeric environments characterizing these micelles are closer to those of a dynamic/liquid, rather than of a static/gel-like flat bilayer.

To obtain quantitative insights we then projected the SOAP *frame*- and *simulation*-averages of these systems along the first two PCs (Fig. 4D), and computed the *d*_SOAP_ matrix (Fig. 4E). The DPPC_273*K*_ and DPPC_293*K*_ assemblies appear relatively close to each other, and separated from the other three systems—while in DPPC_293*K*_ both gel and liquid phases coexist, most of the lipids are gel-like. Similarly, DPC_300*K*_ and SDS_300*K*_ micelles have *d*_SOAP_ ~ 0, appearing close in the scatter plot (Fig. 4D). Interestingly, the liquid DPPC_323*K*_ shows “intermediate” features, being closer (in terms of monomers environment, disorder/reshuffling) to dynamic micelles formed by different monomers (SDS/DPC), than to the DPPC bilayer at lower temperature (Fig. 4D, E).

### Comparing 3D dynamic assemblies

**Fig. 5:**
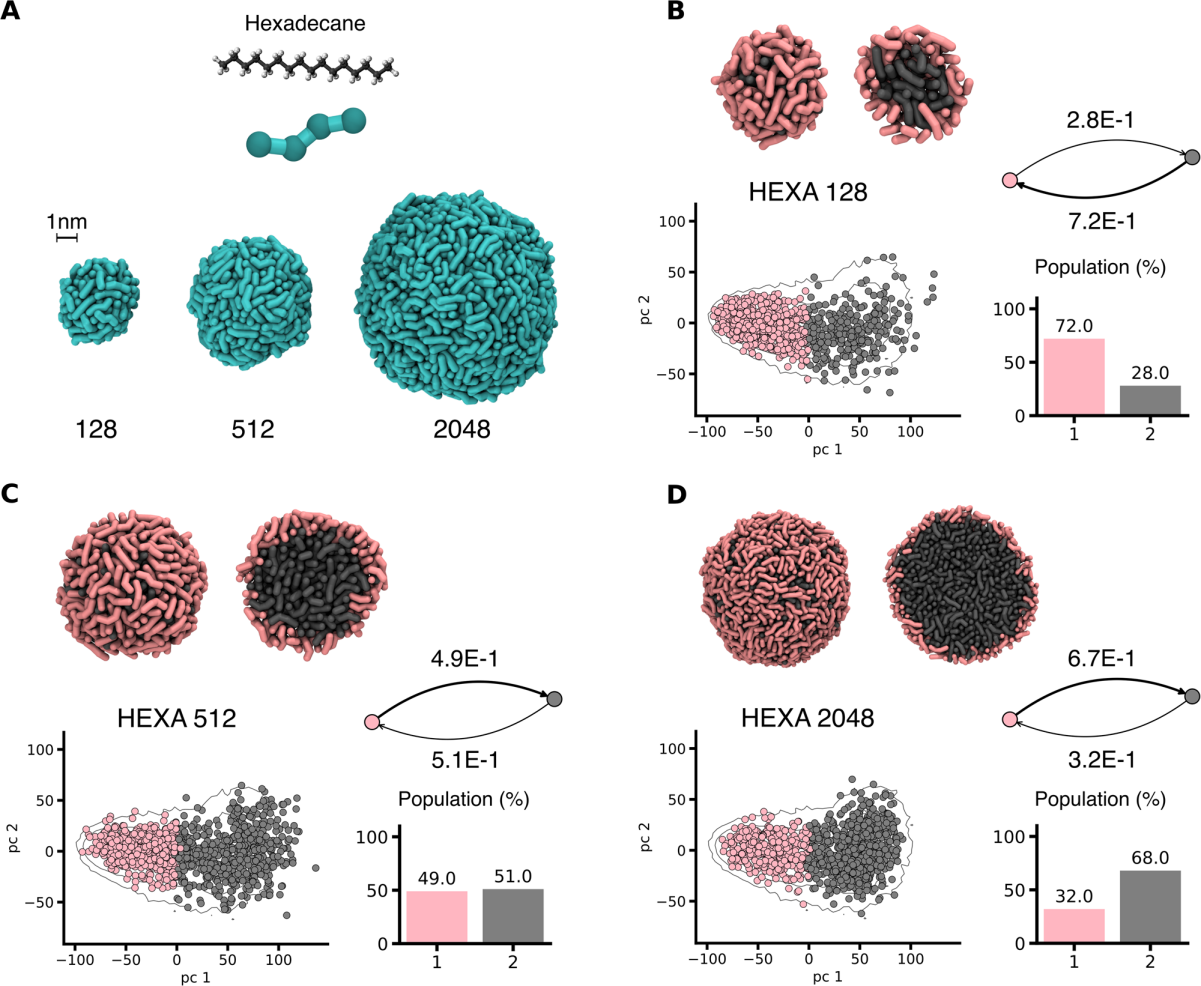
Comparing 3D assemblies. We consider three homogeneous nanoparticle-like structures of different size (composed of a different number of HEXA monomers). **A** Chemical structure and CG model representation of HEXA monomers and growing size nanoparticles. **B**–**D** SOAP + PAMM analysis of the three spherical assemblies formed by a different number of monomers (**B**: 128, **C**: 512 and **D**: 2048 HEXA). Each panel shows (left to right) an equilibrium MD snapshot of the aggregate (entire and in section), the PCA scatter plot of SOAP feature vectors, the molecular motif interconversion graph and the macroclusters population histogram. The colors refer to the molecular environment states detected by PAMM.

We tested this approach also on soft self-assembled 3D systems. In particular, we focused on spherical-shaped nanoparticles. As an example, we chose hexadecane (HEXA), an hydrophobic alkane composed of 16 Carbon atoms, for which we employed a Martini-based CG model. HEXA molecules undergo aggregation forming spherical assemblies (droplets) in water. We tested our method by comparing assemblies of variable size, composed of 128, 512 and 2048 molecules (Fig. 5A). For each assembly, we collected equilibrium MD trajectories in explicit water, and analysed them *via* SOAP + PAMM analysis. Again, we defined one SOAP vector in the center of each monomer. Since the compared systems have different size, we adapted the sampling statistics, considering different number of frames depending on the system size (with fixed sampling frequency of 1 ns^−1^). Therefore, all systems contribute to the global SOAP dataset in equal percentages (see Methods for details).

The comparison of HEXA droplets is reported in Fig. 5B–D. Not surprisingly, the molecular environments do not change radically across different system sizes (scatter plots in Fig. 5B–D). Our analysis identifies two populated motifs, essentially corresponding to the monomers that are on the surface (pink) or in the bulk (gray) of the HEXA assemblies (in general, the larger is the assembly, the more populated is the bulk phase). The analysis also highlights a dynamicity between these two molecular motifs (interconversion between bulk and surface), which varies with the aggregate size in the same way as the ratio between the population of the pink and gray domains. While these cases are rather simple, they show that our analysis provides robust and reasonable results also in the case of 3D assemblies of variable size. This analysis enriches the systems studied in the present work with isotropic 3D assemblies, providing additional data to push the limits of the comparability in the next section.

### A “defectometer” to compare and classify different types of soft dynamic assemblies

As a last step, we processed the simulation data of all the studied systems in a single SOAP + PAMM characterization, assessing to what extent completely different assemblies can be compared to each other. It is worth noting that all self-assembled systems share a common feature, in that they are formed of individual monomers that interact with each other. Having used a single-center per-monomer SOAP definition in each system, we can thus compare all these different assemblies in a common SOAP feature space containing information on the mutual arrangement of their monomers along the equilibrium MD trajectories. We gathered in a single dataset all SOAP vectors computed in the analyses of the previous sections. An identical number of SOAP vectors is considered for each system, to equally weight them, guaranteeing a balanced comparability.

**Fig. 6:**
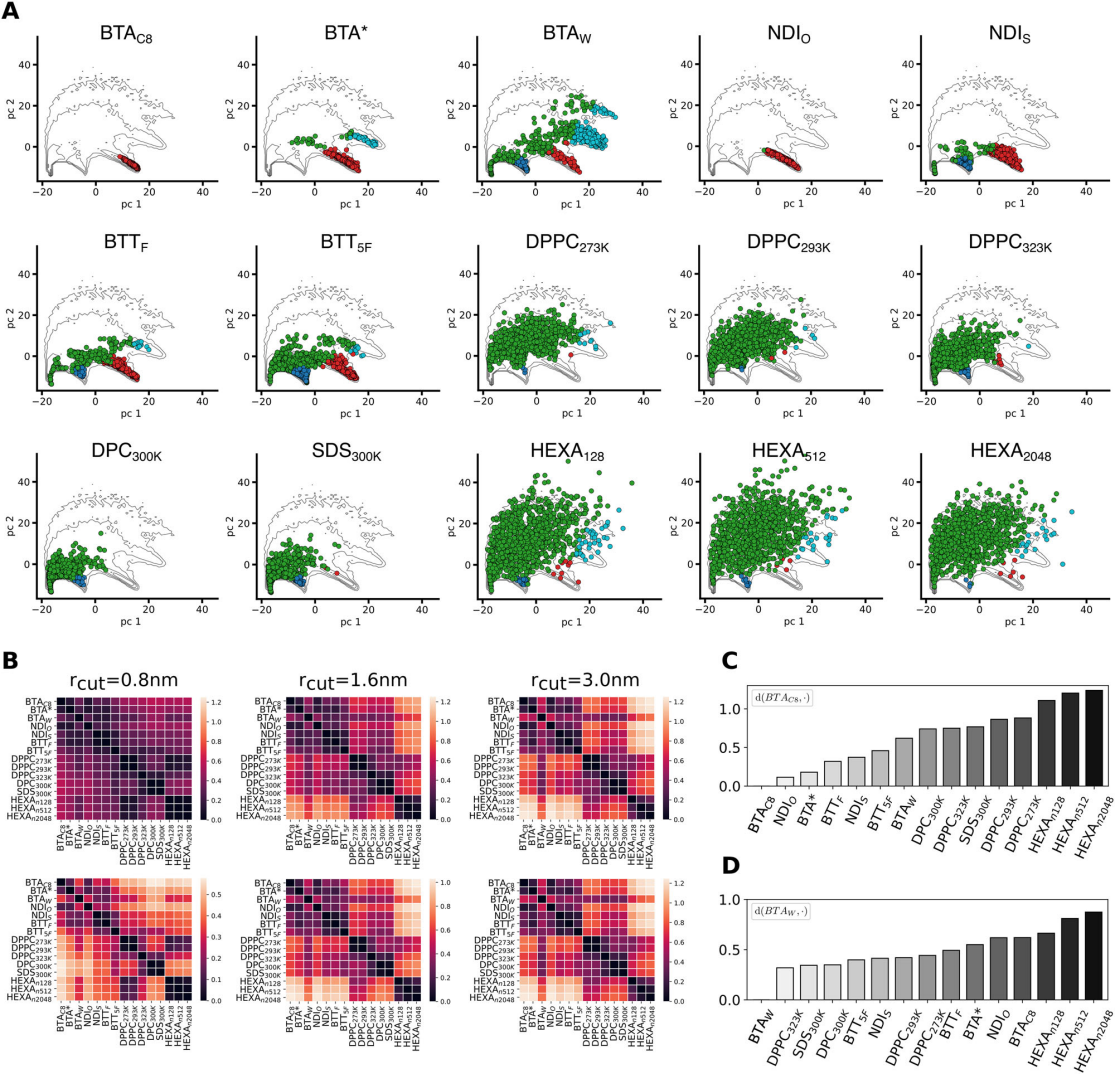
Comparison of different types of soft dynamic supramolecular materials. **A** SOAP + PAMM analysis for all the 15 systems considered in the present work. Each panel reports the PCA scatter plot of SOAP feature vectors relative to a single system projected onto the global SOAP vector dataset (accounting for all considered systems: black contour plot). The colors indicate the molecular motifs detected by the PAMM clustering algorithm. The reported data are obtained using rcut = 0.8 nm in the SOAP analysis (same as in Figs. 1–3). Analogous plots, obtained using larger rcut values (rcut = 1.6 and rcut = 3.0 nm) are shown in Supplementary Figs. 6, 7. **B** Distance *d*_SOAP_ matrices, computed from the SOAP *simulation*-averages using different cutoffs in the SOAP analysis (left: rcut = 0.8 nm, center: rcut = 1.6 nm, right: rcut = 3.0 nm); each matrix is reported twice, using a fixed *d*_SOAP_ scale (top: 0.0 < *d*_SOAP_ < 1.25), or a *d*_SOAP_ scale that is adapted for each case (showing consistent similarity results at all rcut values). **C** Plot of *d*_SOAP_ between BTA_*C*8_ (reference) and all the other assemblies (reported data computed with rcut = 3.0 nm). **D** Same as (**C**) but setting BTA_*W*_ as the reference.

We processed the complete SOAP dataset *via* the described PCA + PAMM workflow. Figure 6A shows the PCA projections of the SOAP vectors for each system, overimposed onto the contour map of the global dataset. Different colors are associated to the five dominant molecular motifs detected *via* PAMM. The systems populate different regions in the SOAP space (Fig. 6A). This comparative analysis provides results compatible with those of previous analyses (e.g., different PCA scatter plots between ordered and disordered fibers). Surprisingly, many systems populate all the identified motifs, suggesting that structural analogies are present, despite the intrinsic diversity of the considered assemblies. Nonetheless, the cluster superposition in the PCA scatter plots hampers the comparison, as relevant dimensions might be hidden. To obtain a more quantitative insight, we turned again to the average SOAP vectors associated to each system. Using the high-dimensional SOAP metric^56^ introduced above, we computed the *d*_SOAP_ matrix comparing the *simulation*-averages associated to each assembly with each other, indicating the similarity and differences between the systems in terms of monomer environments (distance in the SOAP space). An important parameter for the SOAP analysis is the cutoff radius (rcut), which determines the size of the neighborhood considered in characterizing the molecular environment of each SOAP center^7^. In principle, the *d*_SOAP_ scale may change while changing the rcut in the analysis. In order to prove the robustness of our analysis, and given the differences in the assemblies compared herein, we computed the *d*_SOAP_ matrix using three different rcut values: 0.8, 1.6, and 3.0 nm. Shown in Fig. 6B (top panels), the matrices show a reduced *d*_SOAP_ between the various systems when a shorter rcut is used (i.e., the differences between systems fade for rcut = 0.8 nm with respect to rcut = 3.0 nm). The bottom panels of Fig. 6B report the same *d*_SOAP_ matrices of the top panels, with an adapted color scale. Aside from subtle differences—e.g., a slightly lower resolution when a smaller rcut is used—the global picture in terms of similarity between the compared assemblies is consistent in all cases (more details on the effect of changing SOAP + PAMM parameters on the resolution and completeness of the analysis are reported both in the Methods and Supplementary Note 2).

In general, in the *d*_SOAP_ matrices we observe four main dark areas—i.e., that of fibers (1D assemblies), two neighboring/entangled ones for the flat and spherical 2D assemblies, and a third one for the 3D aggregates (bottom-right in the matrix). An interesting exception is the BTA_*W*_ fiber, found more similar to 2D assemblies (DPPC and surfactant micelles) than to the other ordered 1D assemblies. Another interesting system is the DPPC bilayer at 323 K, which is found closer to highly dynamic SDS and DPC micelles rather than to DPPC at lower temperature (293 or 273 K).

**Fig. 7:**
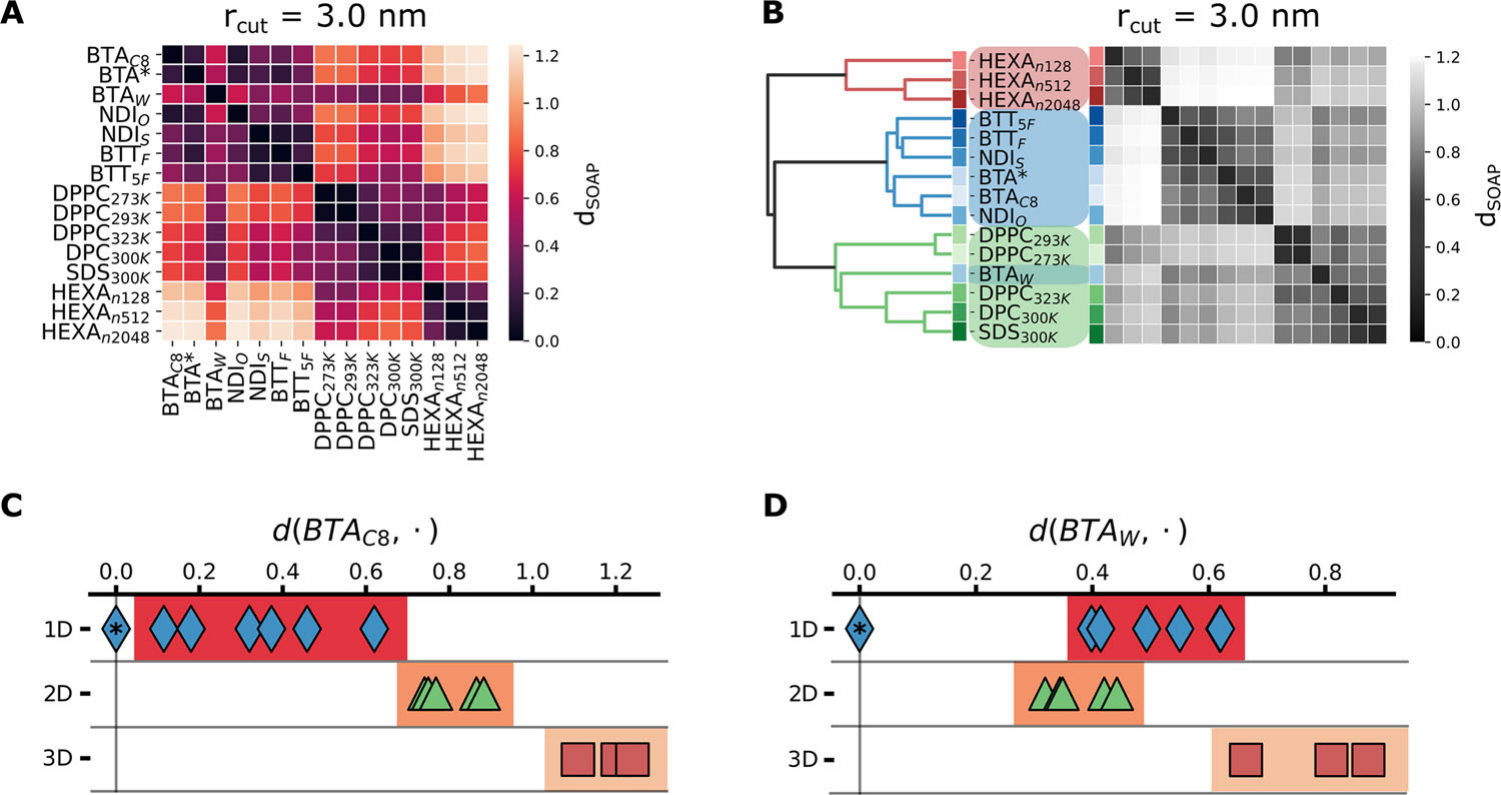
Similarity classification of soft, dynamic supramolecular materials. **A** Distance *d*_SOAP_ matrix (as reported in Fig. 6B) computed from the SOAP *simulation*-averages using a cutoff rcut = 3.0 nm. **B** Hierarchical clustering plot and consequent reshuffling of the *d*_SOAP_ matrix rows and columns, showing grouping of the system according to SOAP similarity. **C** assembly (theoretical) dimensionality as a function of the *d*_SOAP_ distance from the reference BTA_*C*8_. (**D**) same as (**C**) with BTA_*W*_ as reference.

In the *d*_SOAP_ matrix, one can select one assembly and rank all the others with respect to it. For example, selecting the ordered BTA_*C*8_ fiber, the plot of Fig. 6C shows a high similarity (small *d*_SOAP_) with the other 1D ordered assemblies, lower similarity with disordered 1D fibers (e.g., *d*_SOAP_ ~ 0.6–0.7 vs. BTA_*W*_), while *d*_SOAP_ increases further with respect to 2D and 3D assemblies. As anticipated above, an interesting result is obtained for BTA_*W*_ (Fig. 6D). In terms of *d*_SOAP_ ranking, the closer assemblies to this water-soluble, disordered fiber are indeed highly dynamic, planar or spherical 2D assemblies. Surprisingly, all the ordered 1D fibers are less similar to BTA_*W*_ than all the 2D studied systems. This suggests that the solvophobic component of the BTA_*W*_-BTA_*W*_ interactions in water (key factor in controlling the defect formation in such 1D assemblies)^7,8^ can shape a molecular environment in the surrounding of the monomers that is closer to the environment of 2D micelles or liquid-like lipid bilayers than to the environment of ordered BTA variants (e.g., BTA_*C*8_). Noteworthy, this is known to produce a dynamic surface adaptability in this specific fiber that is similar to the surface fluidity seen, e.g., in lipid bilayers^6,10^. The relative *d*_SOAP_ distances in the matrix (computed with rcut = 3.0 nm, Fig. 7A) were then processed *via* a further clustering step (see the Methods section for details), to assess the similarity interconnections among all considered systems. This led to the global dendrogram shown in Fig. 7B, which clearly underlines how the disordered BTA_*W*_ fiber is, for example, closer to the ensemble of 2D assemblies—bilayers and micelles (highlighted in green) -, rather than to that of ordered 1D fibers (in blue)^6,7,10^. This demonstrates that the proposed SOAP-based analysis can provide a handle to classification exceeding the typical ones based on, e.g., the theoretical dimensionality of an assembly, monomer-monomer coordination number, etc., which cope badly with complex and dynamic assemblies. This crucial difference between “standard” human-based classifications and the data-driven method proposed herein is underlined further in Fig. 7C, D. As an example, these plots report the dimensionality of each system as a function of its distance *d*_SOAP_ from the BTA_*C*8_ (C) and BTA_*W*_ (D) systems, showing that a correlation between microscopic similarity and a priori theoretical dimensionality is not obvious when dealing with soft dynamic assemblies as those studied herein.

It is worth noting that such similarity measurements rely solely on data extracted from equilibrium MD simulations, with no major assumption on the structure/features of the studied materials. While this approach is flexible, and can be used to compare in an unbiased way different types of materials, it also creates the important opportunity to classify assemblies based on the molecular environments that populate them, which is a crucial step towards the rational design of supramolecular materials with programmable dynamic properties.

## Conclusion

Rigorous and precise classification of the structure and dynamics of soft supramolecular materials is essential for the rational design of monomers that can self-assemble into supramolecular structures having controllable dynamic behavior. While it is known that the dynamics of such materials originates from defect states that statistically populate them, it is not trivial to design a unified, unbiased, and robust approach to classify soft assemblies based on their “defectivity”.

Here we report an unsupervised, machine-learning approach that allows comparison and classification of soft self-assembled materials (1D supramolecular fibers, 2D, and 3D assemblies) based on the structural dynamics of the internal molecular environments that surround their constituive monomers. We simulate these supramolecular assemblies via molecular models having submolecular (<5 Å) resolution, and analyse their equilibrium MD trajectories *via* a synergistic use of atomic environment descriptors, unsupervised clustering, and similarity/dissimilarity measurements in a high-dimensional feature space. Monomers’ displacement/arrangement data extracted from the MD simulations are translated into relevant information on the molecular arrangements within the assemblies by means of SOAP feature vectors^49^. The high-dimensional data contained in the resulting SOAP spectra are then used to identify dominant monomer clusters/states in the different assemblies *via* unsupervised PAMM clustering (and PCA)^7,51^. The analysis outputs a characteristic fingerprint for each system in terms of the most populated molecular motifs, their structural and dynamical features. A metric defined in the high-dimensional SOAP feature space^55,56^ then provides a quantitative way to compare and classify the studied systems. We show how such data-driven analysis can be used to compare a wide range of supramolecular systems, providing a classification based on the monomeric motifs that emerge from equilibrium MD data, overcoming standard classification approaches based on a priori assumptions on the features of the assemblies (such as, e.g., their ideal/theoretical dimensionality, etc.). The obtained results also demonstrate how, monitoring the monomer transitions between the detected molecular motifs, it is possible to obtain relevant information on the inner dynamics of the assemblies. We can observe how ordered 1D stacked fibers are quite similar with each other, while defected supramolecular polymers (e.g., BTA_*W*_) have a richer and diverse internal structure/dynamics, closer to that of some types of considered lipid bilayers or micelles. This fits well, e.g., with the complex dynamics of the surface of BTA_*W*_ water-soluble fibers^6,61–63^, and with their dynamic adaptivity and structural reconfigurability ^10,64^.

This workflow is powerful for multiple reasons. (i) It does not build on any a priori knowledge on the structure and dynamics of the various assemblies that are compared. (ii) Building on the concept of “defectivity”, it proposes defects as a common ground to compare different materials, a direction that holds a great potential to unify supramolecular materials. (iii) Such a data-driven “defectometer” allows to quantitatively classify dynamic assemblies that are different from each other (e.g., fibers vs. micelles vs. layers vs. nanoparticles). This provides us with a precious tool toward the rational design of self-assembled materials with controllable dynamic properties, which is key to conceive complex systems where multiple assembled entities can effectively communicate with each other in a dynamic way.

## Methods

### Descriptors of atomic environments

Let a generic output of an MD simulation be the ensemble of system conformations sampled at a series of MD timesteps, represented by the atomic coordinates **R**(*t*) of the molecular species in the system at timestep *t*. **R** is a 3*N*-dimensional configuration vector, where *N* is the number of particles. A generic descriptor is a mapping from the 3*N*-dimensional coordinate space to a *D*-dimensional *feature space*, that associates a *feature vector* to each of the sampled conformations **R**(*t*). We aim at a descriptor capable to characterize and compare the atomic/molecular environment surrounding the multiple constituents of a soft, supramolecular system. A first, crucial requirement of this mapping is that it preserves physical symmetries such as permutation, translation and rotation invariance, ensuring that physically equivalent configurations are recognized as such by the descriptor^65^. Other less obvious properties, required for a useful representation of the atomic/molecular environment are e.g., smoothness, additivity and level of locality; SOAP descriptors satisfy all these requirements ^66,67^.

#### Smooth Overlap of Atomic Position (SOAP)

The SOAP^49^ is an atom-centered descriptor, that accurately reproduces density correlation features of many-body systems. SOAP were introduced in ref. ^49^ as new bond-order parameters, able to efficiently account for radial and angular information of the environment that surrounds atoms or molecules^41,49,66^. The SOAP descriptor of a system of *N* particles describes the atomic/molecular surroundings of a selected set of *M* coordinates of the system components, which are referred to as the “centers” of the SOAP vector. These *M* centers can include the position of every single atom of the system, as well as selections or combinations (as e.g., center of geometry/mass) of them. In the following, we comment on the choice of the SOAP center for each of the studied systems, but in general we associate a single SOAP vector per each monomer (see also “Results and discussion”). In this work we have considered only single-specie systems, but the approach is generalizable to multiple species.

The SOAP descriptor is built from the density of neighboring centers *j* that surround the *i*-th center, namely

1 $${\rho }^{i}({{{{{{{\bf{r}}}}}}}})\,=\,\mathop{\sum}\limits_{j}\exp \left[\frac{-| {{{{{{{\bf{r}}}}}}}}\,-\,{{{{{{{{\bf{r}}}}}}}}}_{ij}{| }^{2}}{2{\sigma }^{2}}\right]{f}_{{\mathtt{rcut}}}(| {{{{{{{\bf{r}}}}}}}}\,-\,{{{{{{{{\bf{r}}}}}}}}}_{ij}| ),$$

where a Gaussian function is associated to each neighboring center (located at distance **r** = **r**_*ij*_ from the *i*-th center), to build a smooth density function. The *σ* parameter sets the width of the Gaussian located at the *j*-th neighboring center. The total neighbor density *ρ* = ∑_*i*_*ρ*^*i*^ is retrieved by summing all contributions. The function *f*_rcut_ smoothly goes from 1 to 0 at rcut, so that the environment of each center extends up to a fixed cutoff rcut. Starting from Equation (1), which is intrinsically invariant for the permutation of centers, the SOAP descriptors are defined by incorporating the translation and rotation invariances. This is done in two steps: (i) Eq. 1 is expanded in the basis of orthonormal radial functions *R*_*n*_(*r*) and spherical harmonics ${Y}_{l,m}(\hat{{{{{{{{\bf{r}}}}}}}}})$; (ii) rotational invariance is enforced by building symmetrized combinations of the expansion coefficients^49,66^.

We here employ the second-order SOAP descriptor, also called SOAP power spectrum (in analogy with the Fourier analysis), which can be written as

2 $${\gamma }_{nn^{\prime} l}^{i}\,\propto\, \frac{1}{\sqrt{2l\,+\,1}}\mathop{\sum }\limits_{m\,=\,-l}^{+l}{({c}_{nlm}^{i})}\,{* }\,{c}_{n^{\prime} lm}^{i},$$

where ${c}_{nlm}^{i}$ are the expansion coefficients of the particle density surrounding the *i*th-center. The full SOAP descriptor associated to the *i*-th center is a vector including all contributions from Eq. 2,

3 $${{{{{{{{\bf{p}}}}}}}}}_{i}\,=\,\{{\gamma }_{nn^{\prime} l}^{i}\},$$

where *n* and $n^{\prime}$ range from 1 to ${\mathtt{nmax}}$ and *l* ranges from 1 to ${\mathtt{lmax}}$, setting the dimension *D* of the vector (in cases of multiple species *D* depends also on the number of species)^41,49^. To compute Eq. 2 we used the python package DScribe^68^, using nmax, lmax = 8 and three different cutoff values rcut = 0.8, 1.6, 3.0 nm. The remaining parameters were set to default values of the DScribe library.

To summarize, for a given 3*N*-dimensional configuration vector **R** sampled via CG-MD, we define a set of *M* centers {*i*}, and for each center we compute the SOAP vector {**p**_*i*_}. This generates a dataset of *M* SOAP vectors that describe the structural arrangement of the centers in the selected configuration of the system. Such SOAP vectors represent “local” descriptors, encoding the information on the environments that surrounds each center.

We can also define “global” descriptors, useful for the comparison of different systems: (i) the *frame*-average SOAP descriptor ${\bar{{{{{{{{\bf{p}}}}}}}}}}_{t}\,=\,\{{\bar{\gamma }}_{nn^{\prime} l}^{t}\}$, where each component of the vector is computed as the power spectrum of the density (Eq. 1), after averaging over all the *M* centers, namely ^68^:

4 $${\bar{\gamma }}_{nn^{\prime} l}^{t} \,\sim\, \mathop{\sum }\limits_{m\,=\,-l}^{+l}{\left(\frac{1}{M}\mathop{\sum }\limits_{i}^{M}{c}_{nlm}^{i}\right)}\,{* }\,\left(\frac{1}{M}\mathop{\sum }\limits_{i}^{M}{c}_{n^{\prime} lm}^{i}\right).$$

This gives a global (averaged) picture of the structural features characterizing a specific MD frame. (ii) The *simulation*-average SOAP descriptor,

5 $$\langle \bar{{{{{{{{\bf{p}}}}}}}}}\rangle \,=\,\frac{1}{T}\mathop{\sum }\limits_{t}^{T}{\bar{{{{{{{{\bf{p}}}}}}}}}}_{t},$$

that averages all the *frame*-average SOAP descriptors along the *T* frames collected through the MD simulation. Equation 5 represents a compact global fingerprint for the equilibrium structure of the system under investigation, provided that the *T* frames are sampled at the equilibrium; we used the *frame*-average SOAP to assert similarities between different structures. See further details in Supplementary Figs. 1, 2 and Supplementary Note 1.

#### Comparison of molecular environments

Once SOAP feature vectors are computed, the similarity between the SOAP characterization of different supramolecular structures can be inferred *via* a distance estimation (or kernel function), provided that the dimensionality *D* of the compared SOAP vectors is the same (i.e., using equal nmax and lmax) between the compared systems. We measure the similarity between two SOAP vectors using a linear polynomial kernel ^49,50^,

6 $${{{{{{{\mathcal{K}}}}}}}}(i,j)\,=\,\left({{{{{{{{\bf{q}}}}}}}}}_{i}\cdot {{{{{{{{\bf{q}}}}}}}}}_{j}\right),$$

where **q** = **p**/∣**p**∣ is the unit-normalized SOAP vector. Upon normalization, ${{{{{{{\mathcal{K}}}}}}}}(i,j)$ is equal to 1 if the two molecular environments are exactly superimposed or 0 if no overlap occurs. Since Eq. 6 defines a positive-definite kernel, it naturally induces a metric, for which the distance between two feature vectors is defined as^50,67^

7 $${{{{{{{{\rm{d}}}}}}}}}_{{{{{{{{\rm{SOAP}}}}}}}}}(i,j)\,=\,\sqrt{{{{{{{{\mathcal{K}}}}}}}}(i,i)\,+\,{{{{{{{\mathcal{K}}}}}}}}(j,j)\,-\,2{{{{{{{\mathcal{K}}}}}}}}(i,j)}.$$

Recently, this SOAP-induced metric was employed in ref. ^56^ to compare and classify the structural arrangement of lipid membranes represented *via* different force-fields. We here apply the same approach for the comparison of different supramolecular systems, computing Eq. 7 for the pairs of *simulation*-average SOAP vectors obtained *via* the simulation of each different test case.

The SOAP metric of Eq. 7 fully depends on the high-dimensional information contained in the SOAP spectra, and it provides a classification that is free from prior assumptions on the structural order of the considered systems. The setting of the cutoff radius rcut in the calculation of the SOAP can influence the resulting feature vectors, and few considerations are useful in this sense. The comparative analysis of all the systems, reported in Fig. 6, shows that the choice of a smaller cutoff (rcut = 0.8 nm) can lower the accuracy of the comparison, especially when substantially different systems are compared (see the *d*_SOAP_ matrices in Fig. 6B:top, Supplementary Figs. 6, 7 and 9, and Supplementary Note 2 for additional discussion on this matter). Nonetheless, even a rcut = 0.8 nm (which takes into account only the closest neighbors of the monomers in the SOAP analysis, see Supplementary Fig. 4) appears sufficient to obtain a good characterization of the molecular environment in each system, capturing an analogous pattern of relative distances *d*_SOAP_ with respect to the results obtained with larger rcut (see the rescaled *d*_SOAP_ matrices in Fig. 6B:bottom). We underline that we chose a fixed value of rcut for all the systems, in each of the analysis reported previously. This is made possible by the fact that the studied systems are all described with a similar resolution, namely employing Martini-based CG models. This gives rise to comparable inter-beads distances and facilitates a comprehensive analysis that accounts for the same neighborhood region per each monomer. In principle it should be possible to include in the dataset also simulation results obtained with molecular models at different resolution, provided that the cutoff is differentiated to have a consistent SOAP description across the different models.

### Dimensionality reduction

The SOAP data output of an MD simulation consists of a set of feature vectors (Eq. 3) of dimension *D* (typically large). High-dimensional data, while rich in information, are not well-suited for visualization and classification/clustering, especially with methods based on Euclidean distance. To overcome these limitations a dimensionality reduction method is necessary.

In the realm of available dimensionality reduction algorithms we have opted for the Principal Component Analysis (PCA)^69,70^, a widely used method that was already proven to handle with efficacy high-dimensional SOAP data obtained from high-resolution MD simulations of complex molecular and supramolecular systems^7,54–56^. For such systems, PCA was found to offer a good compromise between efficiency, reliability of the results and affordable computational cost (as shown in Supplementary Fig. 3, discussed in the following).

Briefly, PCA projects the ensemble of SOAP data onto an orthogonal basis set, that best accounts for the variance in the original dataset, namely catching the main directions along which the SOAP feature vectors undergo the larger variations. The first components of this projection contain most of the relevant information, allowing one to retain only few components, thus reducing the dimensionality. We here performed PCA *via* the python class sklearn.decomposition.PCA() from the python library Scikit-learn^71^. After PCA, we chose to keep only three principal components, (if not stated otherwise), as this allows us to maintain more than 90% of the total variance in all our cases (see Supplementary Fig. 3). The PAMM clustering step is performed on 3-dimensional vectors containing the first three PCs of the SOAP feature vectors. For visual representation of the SOAP vectors in the scatter plots (Figs. 1–6) we employed the first two PC projections.

### Building of a training set

Crucial to our comparative analysis is the construction of a “shared” dataset, that comprises SOAP feature vectors from all the individual datasets associated to the systems that are compared. This shared dataset is then used to train the PCA model reducing the dimensionality of the SOAP feature vectors, so that we can proceed with the application of the clustering algorithm and visualization of the data in the PC-plane. The usage of comprehensive data coming from a set of multiple systems allows us to take into account the overall data diversity, and compare the molecular environment across the different systems. To allow for a faster PCA step, we sampled the production trajectories including equal subsets of conformations per each system.

### Unsupervised clustering

Relevant patterns in the SOAP data are detected by means of unsupervised clustering: we used Probabilistic Analysis of Molecular Motifs (PAMM)^51^, a density-based clustering algorithm, specifically developed to partition the SOAP data collected *via* MD simulations. In ref. ^51^ the authors presented the complete workflow for the algorithm. PAMM takes as input a set of *N* feature vectors, that represent local or global SOAP descriptors. As detailed earlier, in our case the algorithm processes the three-dimensional projections of SOAP vectors on the first three PCs. Then, the Probability Distribution Function (PDF) of these dimensionally reduced SOAP vectors is estimated by means of a Kernel Density Estimation (KDE), built from a multivariate anisotropic Gaussian function in the 3D space. In order to mitigate the computational load of KDE, the PDF estimation is done on a grid of *N*_grid_ ⊂ *N* points, selected non-uniformly through a farthest point sampling method. After the PDF is accurately estimated a density-based clustering algorithm, based on Gaussian Mixture Modeling (GMM), associates each different local maximum in the PDF to a cluster. Once this analysis is completed we obtain the so-called Probabilistic Motifs Identifiers (PMIs) (as the molecular motifs were called in the original reference)^51^ that characterize the features of the system under consideration. The robustness of the clustering analysis (in terms of identified microclusters) is assessed by means of a bootstrapping procedure^51^. During this step, the SOAP vectors are resampled generating several (*N* = 73) independent samples of SOAP vectors (sub-datasets).The clustering procedure (KDE and GMM steps) is then performed on each resampled sub-dataset, and the resulting clusters are compared to those obtained using the original (non-resampled and complete) dataset. This comparison results in a stability matrix, which measures how robust the detected clusters are. From this matrix we can build a hierarchical tree (dendrogram) that indicates the adjacency of the obtained microclusters (or the monomer microstates) in the SOAP space. The analysis is performed on snapshots collected along equilibrium MD trajectories, keeping track of the microclusters to which each individual monomer belongs to at each timestep. Thus, this analysis allows us to retrieve information on how structurally similar/different and how dynamically interconnected (i.e., how quickly/slowly monomers exchange between the microstates) the detected microclusters are. The dendrogram of Fig. 1C describes how the microclusters can be condensed in macroclusters (based on their structural/dynamic adjacency), obtaining a coarse-grained version of the analysis (see also Supplementary Fig. 5). All PAMM clustering analyses were conducted using the original PAMM algorithm (Available online at https://github.com/cosmo-epfl/pamm) modified with a tailored Python3 wrapper for handling the different analysis steps and post-processing.

#### Hierarchical clustering

Hierarchical clustering generally refers to clustering methods that fracture datasets into subsets according to a selected measure of (dis)similarity. In our work we employed the SOAP metric *d*_SOAP_ to merge pairs of clusters in higher-level groups, until the hierarchy of all the systems is completed. This results in the tree-like plot or dendrogram reported in Fig. 7B, showing the connectivity among the systems. The hierarchical clustering step was performed using the open source Python library Scikit-learn sklearn.cluster.AgglomerativeClustering() and choosing as linkage method single.

### Molecular dynamics simulations

Our analyses build on sampling conformations/transitions during equilibrium MD simulations of the different assembled systems that are studied herein. In general, since in these analyses we reconstruct the internal dynamics and the equilibrium microstates present in these assemblies, a prime requisite for the robustness of the obtained results is that the monomer conformations and transitions are statistically well-sampled, in order to obtain a reliable representation of the equilibrium for each considered assembly (compatibly with the approximations and limits of the molecular models that are used). For this reason, in all systems considered herein, the length of the MD simulations is in the order of microseconds, ensuring that the equilibrium ensemble of each system is well represented by the collected conformational data. In the following we describe the MD approach and sampling protocol that we adopted to ensure this condition for each individual systems simulated herein.

All the simulations presented in this work were performed using the MD package GROMACS^72^, version 2018.6, from the setup of the simulation box, to the equilibration and production runs. We adopted CG descriptions for each system, built *via* the Martini scheme^58^, with explicit solvent description. The parametrization of the monomer models was conducted following the literature data (where available), using the standard, non-polarizable Martini force-field^58^ (version 2.2). Details per each system are provided in the following. All simulations were performed using Periodic Boundary Conditions (PBC) to limit the finite-size effects, also allowing to simulate portions of infinite aggregates or membranes. To prepare the CG model systems for MD in equilibrium conditions we performed CG-MD equilibration runs of the order of μs in NPT conditions, starting from energy-minimized system conformations. We then proceeded with the production CG-MD runs, to collect the statistics of equilibrium configurations processed in our analyses; all our systems where sampled every 1 ns of CG-MD. For all the CG-MD runs (equilibration and production) we used a 20 fs timestep, an interaction cutoff (1.1 nm) where the non-bonded potentials are truncated and shifted (consistently with the Martini scheme). The Verlet neighbor list scheme^73^ is employed to reduce the load of calculating pair interactions. The temperature was maintained through the V-rescale thermostat^74^, set at 300 K for all the simulations, except the cases where a different temperature is stated (i.e., for lipid membranes) with a coupling constant of 1.0 ps. The pressure was maintained *via* Berendsen barostat^75^, set at 1 atm with a coupling constant of 2 ps. Isotropic scaling of the simulation cell is adopted when finite aggregates are simulated (e.g., the HEXA clusters), while semiisotropic scaling (decoupling *x*/*y* from *z*) is adopted when a portion of an infinite aggregate is considered (crossing the PBCs along one or two axes), as for fibers and membranes.

In the following, we report the model references and the specific simulation setup for each system studied.

#### Supramolecular polymers (1D assemblies)

The supramolecular polymers studied in this work belong to three families, characterized by a specific chemical structure of their monomer functional core. For each family we considered monomer variants:

- 1,3,5-Benzenetricarboxamides (BTA) monomers, already extensively studied in the literature^6,7,76^. We considered three different variants: a water-soluble BTA_*W*_ (Fig. 1A), an organic solvent (e.g., octane) soluble BTA_*C*8_ (Fig. 1B), and an intermediate case, BTA^*^ (Fig. 1C), obtained from BTA_*C*8_ by artificially changing the inter-monomer interaction. The parametrization used for the three monomer models was the same as in ref. ^7^ and the solvents, water and octane, were parametrized according to Martini standards^58^. For the calculation of SOAP vectors we associated a single center for each BTA monomer, namely the center-of-geometry (COG) of the monomer core (the three central beads in Fig. 1), which was proven sufficient to describe the structural dynamics of such supramolecular fiber ^7^.
- Core-substituted naphthalene diimide (NDI)^59^ monomers (Fig. 2A); we studied two different variants of NDIs, by changing the substituent atom on the side of the core structure. Following ref. ^59^ we considered a substituted Oxygen (NDI_*O*_, Fig. 2A) and Sulfur (NDI_*S*_, Fig. 2B). The parametrization of the two monomer models was that of ref. ^59^. In both cases the solvent was cyclohexane, parametrized according to Martini standards^58^. Also for the analysis of NDI dynamics we selected the COG of the monomer cores (which correspond to the central pink bead in the central arrangement of five pink beads in Fig. 2A, B) as SOAP center.
- Benzotrithiophene (BTT)^60^ monomers; we studied two variants of these planar, water-soluble monomers, changing the three amino-acids attached to the aromatic core of the molecule. As in ref. ^60^ we used L-phenylalanine for the first variant (BTT_*F*_, Fig. 2C), and pentafluoro-L-phenylalanine for the other (BTT_5*F*_, Fig. 2D). Both monomers present octaethylene glycol side-chains that impart water-solubility to the compound. The CG parametrization of these monomers was the same of ref. ^60^. The solvent used for these BTT polymers was water, parametrized according to Martini standards (Martini water^58^). The COG of the monomer cores (computed amongst the 3 central pink beads in Fig. 2C, D) was employed as SOAP center.

For all the seven fiber-systems we have built an ordered, one-dimensional stack that crosses the PBCs along the principal axis of the aggregate, so that the monomers at the extremes are in contact with each other mimicking and a infinite-fiber portion. Each pre-stacked aggregate was then equilibrated, and a CG-MD production run of about ~2 μs was performed to sample the structural dynamics in equilibrium conditions.

#### Micelles and membranes (2D assemblies)

The lipid molecule selected as reference case for two-dimensional aggregates was the dipalmitoylphosphatidylcholine phospholipid (DPPC, Fig. 4A) for which homogeneous membranes at three different temperatures (273, 293, and 323 K, Fig. 4B) were prepared. In this work we utilized the CG Martini parametrization adopted in ref. ^56^, where SOAP + PAMM characterization of this phospholipid membrane, modeled using different descriptions, is performed. As test cases for the micelle aggregates we selected two surfactant molecules, namely Dodecylphosphocholine (DPC), and Sodium-dodecylsulfate (SDS); both parametrized according to the standard Martini force-field^58,77^. The explicit solvent used for these lipid and surfactant systems was Martini water. The equilibrated structure of the surfactant micelles was obtained *via* spontaneous self-assembly from dispersed monomers, to obtain a size near the normal size distribution at our conditions. The adopted equilibration procedure for the bilayer models is described in ref. ^56^ (which is the minimization and equilibration protocol given by CHARMM-GUI^78^). For each system we performed production CG-MD runs of 1 μs of simulation time. For DPPC, DPC and SDS molecules we employed a single-center approach for the SOAP vector calculation, choosing the CG-bead that represents the head of the amphiphilic molecules as SOAP center (i.e., the “PO4” (DPPC), “PO4” (DPC) and “SO3” (SDS) beads in Fig. 4A). The results of an extra SOAP + PAMM analysis, limited to DPC and SDS systems are reported in Supplementary Fig. 8.

#### Spherical nanoparticles (3D assemblies)

The molecule chosen as representative case for spherical aggregates is the alkane hydrocarbon Hexadecane (HEXA); we parametrized this molecule following the Martini force-field standards^58^. The explicit solvent used for these simulations was Martini water. Three aggregate structures of different size (128, 512, and 2048 monomers) were constructed via 2 μs of CG-MD equilibration run, starting from randomly dispersed monomers in water. During such CG-MD stage the monomers quickly self-assemble and equilibrate forming the spherical structures represented in Fig. 5A). Production runs of different length were carried out to gather the data for the analysis of the equilibrium structures, collecting the same number of SOAP vectors per each systems, independently of their different sizes. Namely, the 128 monomer system was simulated for 2 μs, the 512 monomer system for 0.5 μs and the 2048 monomer system for 0.125 μs. As center for the SOAP representation we chose the COG of the two central beads in the CG model of HEXA.

## Supplementary information

Supplementary Information

## Data Availability

Details on the molecular models and MD simulations, and additional MD data are provided in the “Methods” section and in the Supplementary Information. Computational materials and data pertaining to the study conducted herein are available at: 10.5281/zenodo.6822330. Other information needed is available from the corresponding author upon request.
