# Supplementary material for: Classifying soft self-assembled materials via unsupervised machine learning of defects
Source: Commun Chem. 2022 Jul 14;5:82. doi: 10.1038/s42004-022-00699-z (PMC9814741; doi:10.1038/s42004-022-00699-z)
Supplement: Supplementary file 1 — Supplementary Information [file 42004_2022_699_MOESM1_ESM.pdf]

Supplementary Information for: Classifying soft self-assembled materials via unsupervised machine learning of defects

Andrea Gardin[1], Claudio Perego[2], Giovanni Doni[2], and Giovanni M. Pavan[1,2,*]

[1]Department of Applied Science and Technology, Politecnico di Torino, Corso Duca degli Abruzzi 24, I-10129 Torino, Italy
[2]Department of Innovative Technologies, University of Applied Sciences and Arts of Southern Switzerland, Polo Universitario Lugano - Campus Est, Via la Santa 1, CH-6962 Lugano - Viganello, Switzerland
[*]giovanni.pavan@polito.it

Supplementary Note 1: Schematic workflow

Comparison analyses presented in the main text follow a custom recipe that can be schematically summarized in Supplementary Figure 1. In Supplementary Figure 2A-C the treatment of the data and its transformations from the MD trajectory conformations to the clustering of molecular motifs is presented with more detail; this scheme holds both when treating "local" SOAP descriptors associated to a single centre, and "global" SOAP descriptors like the *frame*-average and the *simulation*-average (defined in the Methods section in the main text).

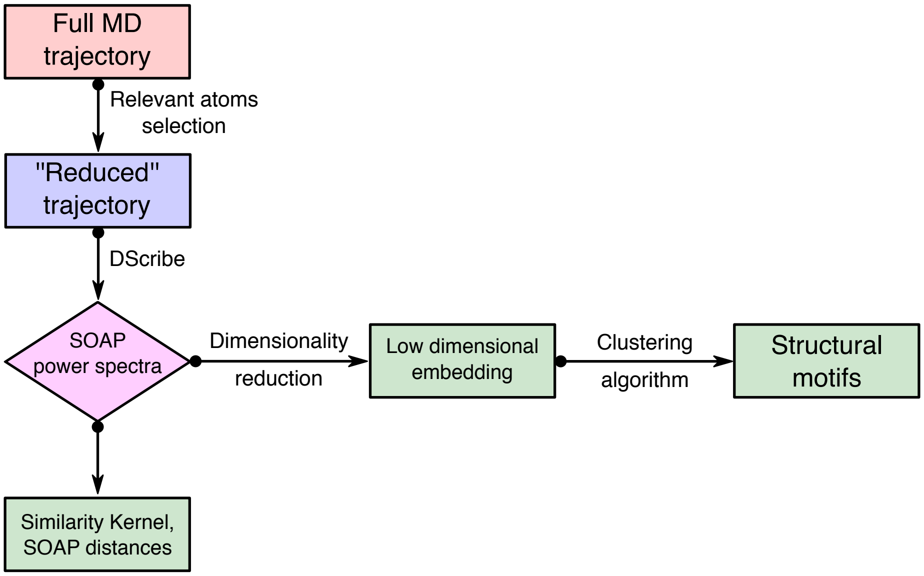

Supplementary Figure 1: Step-by-step schematic workflow of the comparative analysis method.

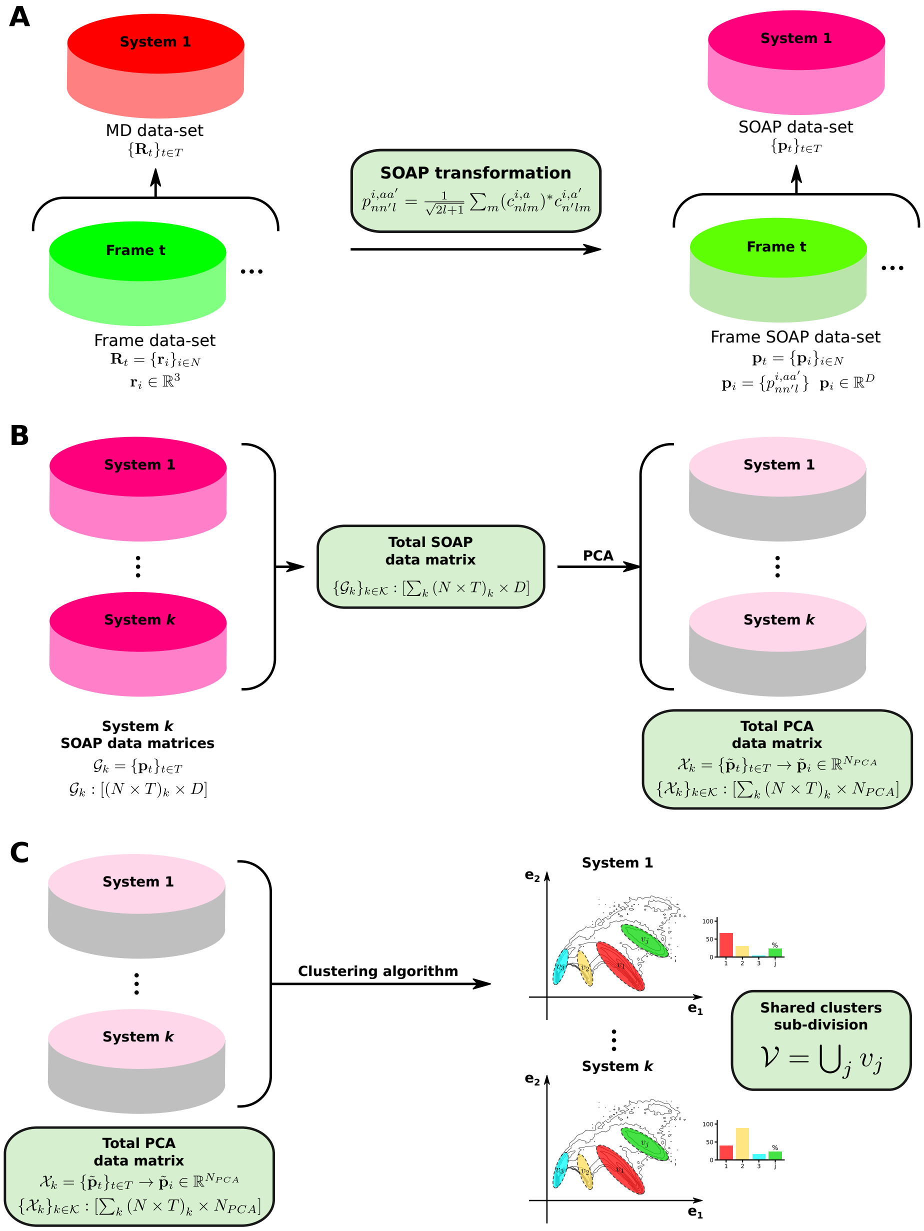

Supplementary Figure 2: In-depth schematic workflow of the comparative analysis method: (A) MD trajectory data representations in terms of SOAP feature vectors for for each chosen "center" of a system and for each chosen frame. (B) Merging of SOAP datasets from multiple systems and computation of the PCA over the resulting merged data-set. (C) From the PCA data-set a clustering algorithm is applied to identify the molecular motifs and characterize the similarities among the systems.

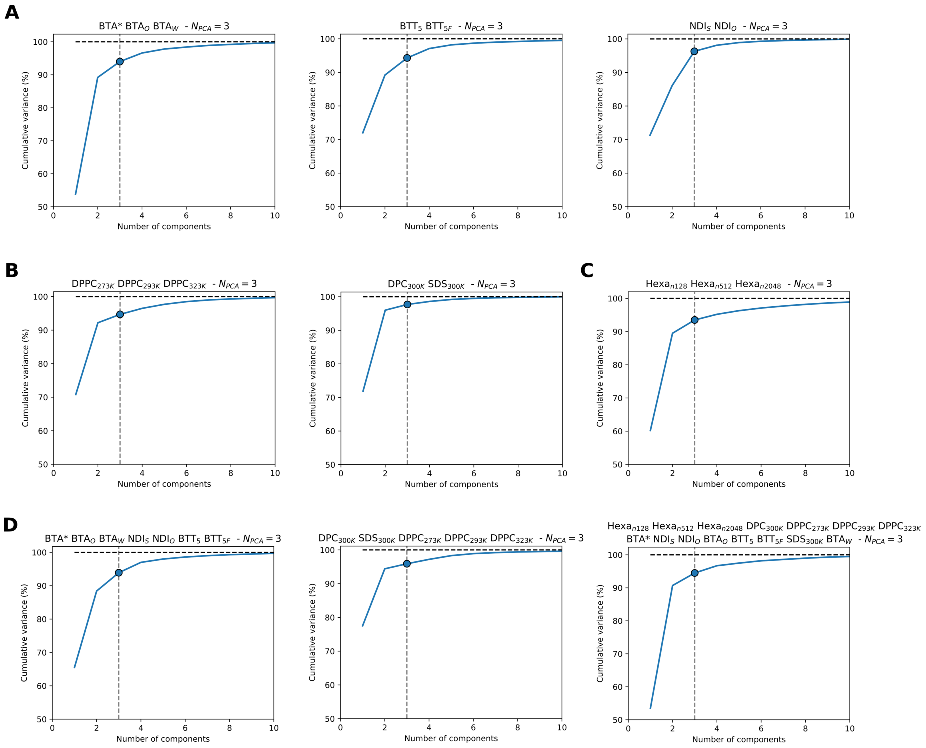

Supplementary Figure 3: PCA variance for all the systems studied in the main text, as a function of the number N_{PCA} of principal components retained in the dimensionality reduction. The variance corresponding to $N_{PCA} = 3$ is highlighted. (A) Supramolecular polymers, (B) micelles and lipid membranes, (C) nanoparticles, (D) joint dataset of respectively (from left to right) all the supramolecular polymers, all the micelles and lipid membranes, and all the systems combined. The systems included in the analysed dataset are listed at the top of each plot.

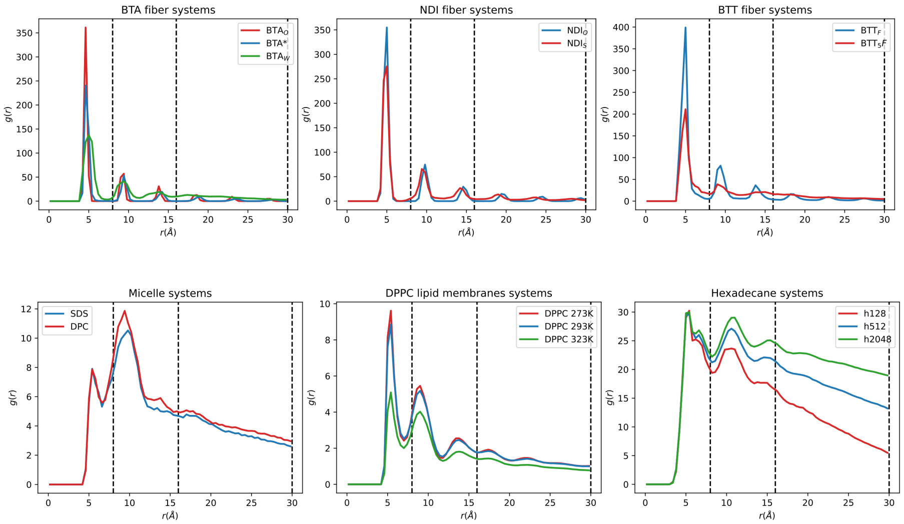

Supplementary Figure 4: Radial distribution functions for each system studied in this work (solid lines). The dashed vertical lines indicate the three different `rcut` values employed in our analyses, namely 0.8, 1.6, 3.0 nm.

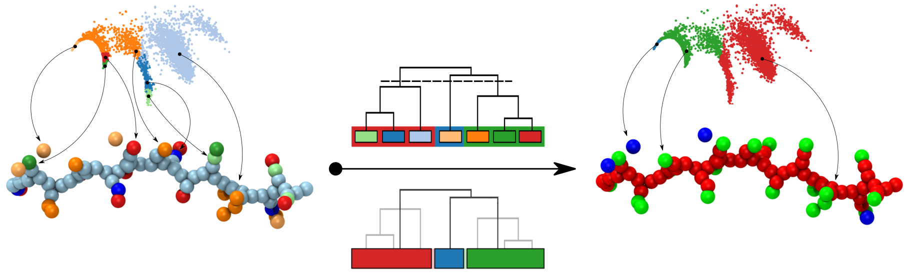

Supplementary Figure 5: Merging of micro-cluster representation into macro-clusters for the BTA_W system. The PCA scatter-plot coloured according to the micro-cluster classification is reported on the left, with a representative snapshot indicating the configuration of the monomer centers associated to each of the states. The dendrogram in the center illustrates the hierarchical clustering step that leads to the simplified classification on the right (see the Methods section in the main text)

Supplementary Note 2: Systems comparison at `rcut` = 1.6, 3.0 nm

Following the definition of the SOAP[1] descriptor, changing the cutoff `rcut` directly changes the fingerprint of the atomic environment surrounding each center in the system. This change affects more significantly the high-dimensional aggregates, where the coordination of centers scales increasing with the cutoff radius, as compared to one-dimensional supramolecular polymers (see also Supplementary Figure 4 for reference). The overall fingerprints obtained via SOAP descriptors appear to be more rich in information, since every small difference in the coordination shells leads to a different SOAP feature vector. In this sense at higher cutoff radius we obtain a larger data variance, after dimensional reduction, corresponding to the inclusion of higher coordination shells in the descriptor definition. In Supplementary Figure 6 the areas corresponding to the green and red clusters reflect respectively the surface of the membranes/micelles and the core of the nanoparticles; In Supplementary Figure 7 the areas corresponding to the blue and red clusters reflect respectively the core of the nanoparticles.

Given that the "local"-SOAP feature vectors account for all these little differences as they should, the "global"-SOAP descriptor still carries an averaged fingerprint of the whole structural aggregates, moreover, translating it to mutual distances (using the definition of SOAP metrics) we observe that the trend is more or less conserved along the cutoff values (Figure 6 of main text).

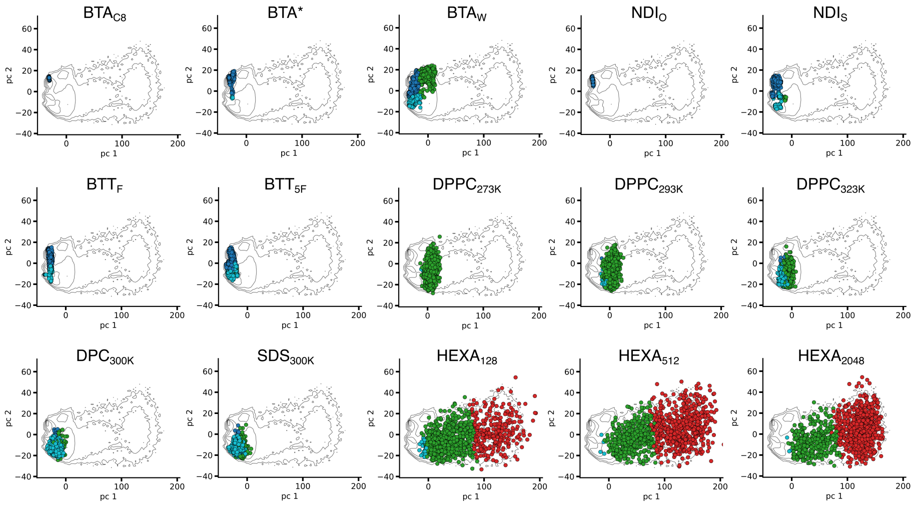

Supplementary Figure 6: Comparison at `rcut` = 1.6 nm. Each panel reports the PC scatter plot of SOAP feature vectors relative to a single system (in colour) superimposed over the SOAP feature vectors of the global dataset (black contour plot). The colours indicate the molecular motifs detected by the PAMM clustering algorithm.

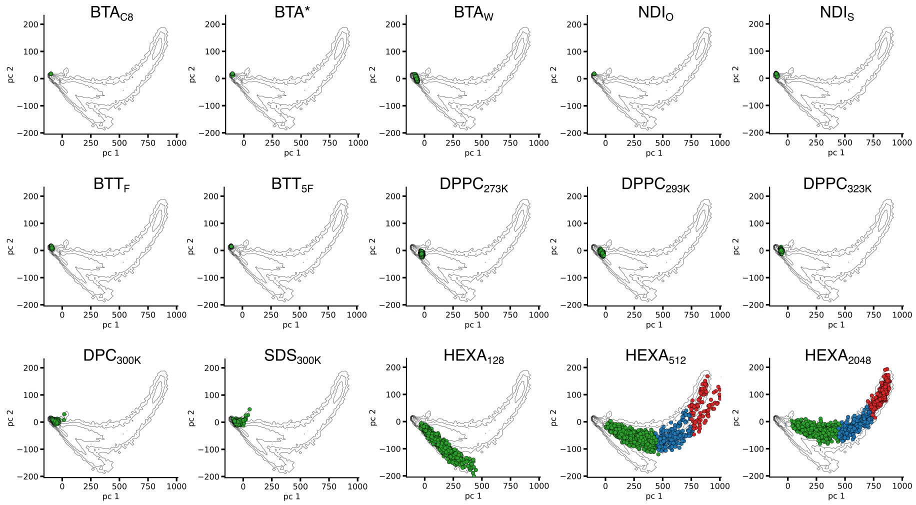

Supplementary Figure 7: Comparison at `rcut` = 3.0 nm. Each panel reports the PC scatter plot of SOAP feature vectors relative to a single system (in colour) superimposed over the SOAP feature vectors of the global dataset (black contour plot). The colours indicate the molecular motifs detected by the PAMM clustering algorithm.

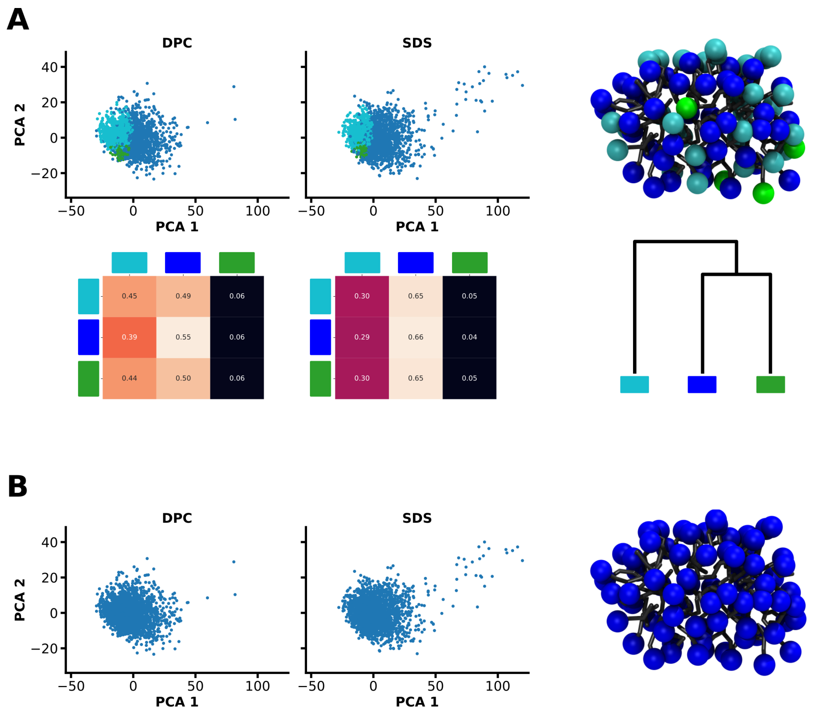

Supplementary Figure 8: SOAP+PAMM analysis applied to the dataset composed by the two different micellar systems (SDS and DPC). The PCA projection along the first two components does not show a particularly interesting "fingerprint", as confirmed by the clustering algorithm. Looking at the molecular microstates detected by the PAMM algorithm (see Ref. 2 for the details of the method) minor differences in the SOAP environments are identified, due mostly to variations in the mutual organization of monomers (A). This distinction is hardly to be attributed to a different phase behavior. This is confirmed by the statistical analysis of the dynamic between these micro-states, which exhibits fast reshuffling between these molecular motifs, so that they can be identified into a single macro-state (B).

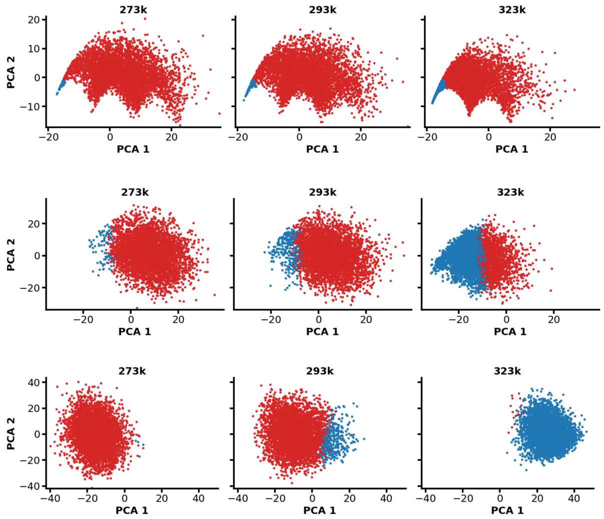

$(r_{cut} = 8\AA)$	$DPPC_{273K}$	$DPPC_{293K}$	$DPPC_{323K}$
Red	0.993	0.983	0.831
Blue	0.007	0.016	0.169

$(r_{cut} = 16\AA)$	$DPPC_{273K}$	$DPPC_{293K}$	$DPPC_{323K}$
Red	0.985	0.943	0.665
Blue	0.015	0.056	0.335

$(r_{cut} = 30\AA)$	$DPPC_{273K}$	$DPPC_{293K}$	$DPPC_{323K}$
Red	< 0.999	0.946	0.004
Blue	> 0.001	0.054	0.996

Supplementary Figure 9: SOAP+PAMM analysis results for the DPPC lipid membranes systems using different `rcut` values of 0.8, 1.6, 3.0 nm. The data shows how `rcut` in the SOAP descriptor calculation affects the separation of the two environments belonging to the liquid and gel phases. A large cutoff radius is needed in order to catch enough detail to better differentiate the local environments.

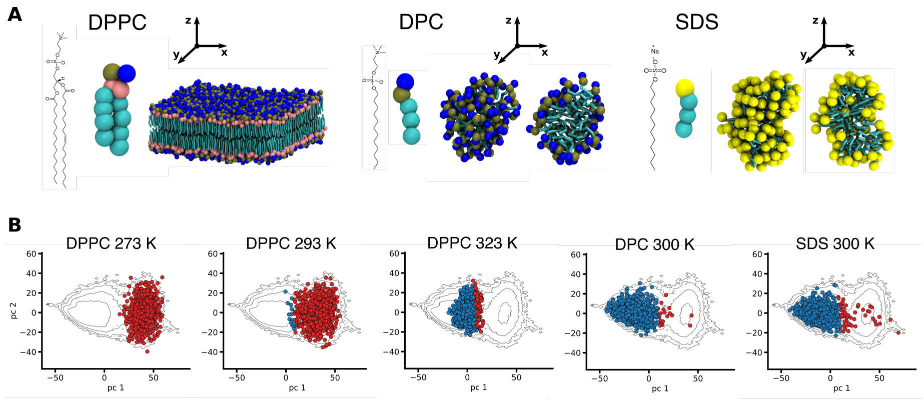

Supplementary Figure 10: SOAP+PAMM analysis for the 2D supramolecular aggregates (DPPC, DPC and SDS) combined. In the PCA scatterplots the micelles systems position themselves closer to the liquid DPPC membrane (at temperature of 323 K), indicating higher similarity of the local environments populated by these systems, although a quantitative difference is signaled by the comparison of averaged SOAP data and by the calculation of the relative SOAP distances.

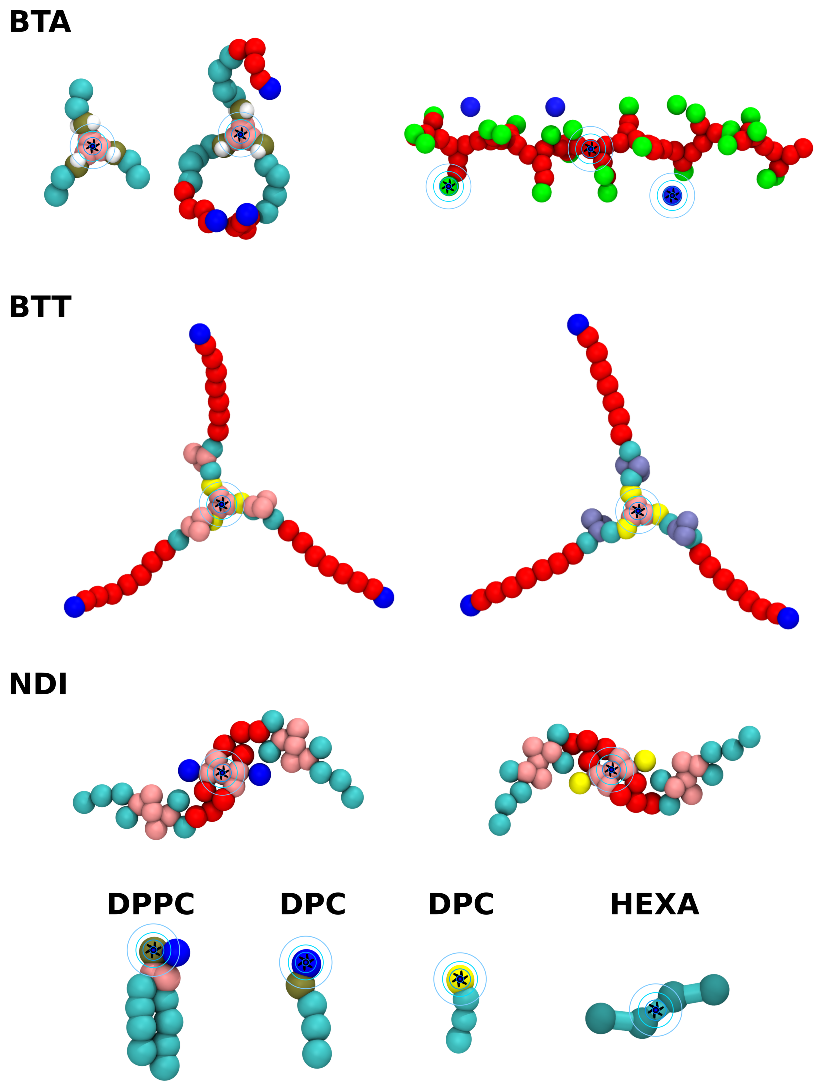

Supplementary Figure 11: SOAP center defined in the analysis, indicated as a black star for each of the studied monomer models. The light blue circles indicate the different choices of `rcut` used in this work, namely 0.8, 1.6, 3.0 nm.

References

[1] Bartók, A. P.; Kondor, R.; Csányi, G. *Phys. Rev. B* **2013**, *87*, 184115.

[2] Gasparotto, P.; Meißner, R. H.; Ceriotti, M. *J. Chem. Theory Comput.* **2018**, *14*, 486–498.
